# Phenotypic and genomic characterization of *Pseudomonas aeruginosa* isolates recovered from catheter-associated urinary tract infections in an Egyptian hospital

**DOI:** 10.1099/mgen.0.001125

**Published:** 2023-10-30

**Authors:** Mohamed Eladawy, Jonathan C. Thomas, Lesley Hoyles

**Affiliations:** ^1^​ Department of Biosciences, School of Science and Technology, Nottingham Trent University, Nottingham, UK; ^2^​ Department of Microbiology and Immunology, Faculty of Pharmacy, Mansoura University, Mansoura, Egypt

**Keywords:** multilocus sequence typing, antimicrobial resistance, biofilm formation, virulence factors, megaplasmid

## Abstract

Catheter-associated urinary tract infections (CAUTIs) represent one of the major healthcare-associated infections, and *Pseudomonas aeruginosa* is a common Gram-negative bacterium associated with catheter infections in Egyptian clinical settings. The present study describes the phenotypic and genotypic characteristics of 31 *P *. *aeruginosa* isolates recovered from CAUTIs in an Egyptian hospital over a 3 month period. Genomes of isolates were of good quality and were confirmed to be *P. aeruginosa* by comparison to the type strain (average nucleotide identity, phylogenetic analysis). Clonal diversity among the isolates was determined; eight different sequence types were found (STs 244, 357, 381, 621, 773, 1430, 1667 and 3765), of which ST357 and ST773 are considered to be high-risk clones. Antimicrobial resistance (AMR) testing according to European Committee on Antimicrobial Susceptibility Testing (EUCAST) guidelines showed that the isolates were highly resistant to quinolones [ciprofloxacin (12/31, 38.7 %) and levofloxacin (9/31, 29 %) followed by tobramycin (10/31, 32.5 %)] and cephalosporins (7/31, 22.5 %). Genotypic analysis of resistance determinants predicted all isolates to encode a range of AMR genes, including those conferring resistance to aminoglycosides, β-lactamases, fluoroquinolones, fosfomycin, sulfonamides, tetracyclines and chloramphenicol. One isolate was found to carry a 422 938 bp pBT2436-like megaplasmid encoding *OXA-520*, the first report from Egypt of this emerging family of clinically important mobile genetic elements. All isolates were able to form biofilms and were predicted to encode virulence genes associated with adherence, antimicrobial activity, anti-phagocytosis, phospholipase enzymes, iron uptake, proteases, secretion systems and toxins. The present study shows how phenotypic analysis alongside genomic analysis may help us understand the AMR and virulence profiles of *P. aeruginosa* contributing to CAUTIs in Egypt.

## Data Summary

The draft genome sequences included in the study are available under BioProject PRJNA913392. Supplementary data and material associated with this article are available from figshare at https://figshare.com/projects/Phenotypic_and_genomic_characterization_of_Pseudomonas_aeruginosa_isolates_recovered_from_catheter-associated_urinary_tract_infections_in_an_Egyptian_hospital/156639. Supplementary tables and figures are available with the online version of this article.

**Impact Statement**

In-depth genotypic and phenotypic characterization of clinical pathogens contributing to the antimicrobial resistance burden in low- and middle-income countries is often not possible due to limited resources. Here we characterize 31 *Pseudomonas aeruginosa* isolates recovered from catheter-associated urinary tract infections in an Egyptian hospital over a 3 month period. We demonstrate that, even with this small number of isolates, genetically diverse isolates and high-risk clones (i.e. ST357 and ST773) of *P. aeruginosa* are present in this clinical setting, and that novel resistance determinants can be readily detected in genomic data. In addition, we provide the first report of a pBT2436-like megaplasmid in a clinical *P. aeruginosa* isolate recovered in the Middle East and North Africa region. Our data will be invaluable in furthering the design of diagnostics and therapeutics for the treatment of *P. aeruginosa* infections in Egypt, and demonstrate that continuous monitoring and surveillance programmes should be encouraged in the country to track the emergence of new (high-risk) clones and to identify novel resistance determinants.

## Introduction

Urinary tract infections (UTIs) are among the most common bacterial infections that affect humans during their life span. They account for over 40 % of all healthcare-associated infections (HAIs) [1]. UTIs can be classified as uncomplicated or complicated depending on the site of infection and disease progress [2]. Urinary tract catheterization is a common practice that predisposes the host to complicated UTIs [3]. Instillation of a catheter in the urinary tract may cause mucosal-layer damage, which disrupts the natural barrier and allows bacterial colonization [4].

*Pseudomonas aeruginosa* is an opportunistic pathogen that causes severe UTIs that are difficult to eradicate due to high intrinsic antimicrobial resistance (AMR) and the bacterium’s ability to develop new resistances during antibiotic treatment [5]. UTIs caused by multidrug-resistant (MDR) *P. aeruginosa* were associated with an overall mortality of 17.7 % at 30 days and 33.9 % at 90 days after admission to a Spanish hospital, and account for 7–10 % of nosocomial UTIs worldwide [6]. In 2021, approximately one-third of *P. aeruginosa* isolates (31 %, *n*=22479) reported for the European Union/European Economic Area (EU/EEA, excluding the UK) were resistant to at least one antimicrobial group under surveillance (piperacillin–tazobactam, fluoroquinolones, ceftazidime, aminoglycosides and carbapenems) [7]. Resistance to two or more antimicrobial groups was found in 17.9 % of all isolates [8]. Although there was a decrease in AMR associated with *P. aeruginosa* for carbapenems, fluoroquinolones and aminoglycosides in the EU/EEA region between 2017 and 2021, resistance remained high in eastern and south-eastern parts of Europe [8].

The World Health Organization (WHO) named *P. aeruginosa* as a target of the highest priority for the development of new antibiotics [9]. Infections caused by MDR *P. aeruginosa* were associated with a 70 % increase in cost per patient [10]. According to the Centers for Disease Control and Prevention (CDC), more than 32 600 cases of HAIs were caused by MDR *P. aeruginosa* in the USA in 2017, which resulted in 2700 deaths and USD $767 million of estimated healthcare costs [11]. In Egypt, mono-microbial infections represented 68.5 % of CAUTIs, while poly-microbial infections represented 31.43 % of catheterized patients admitted in 2021. Moreover, the prevalence of biofilm-dependent CAUTIs was ~82 %. The majority (81.25 %) of patients with catheters inserted for ≤14 days suffered from mono-bacterial colonization inside the catheter, and 42.11 % of patients with catheters inserted for 1 month had poly-microbial colonization [12].

There is extensive variation in the epidemiology of MDR *P. aeruginosa* in the Middle East and North Africa (MENA) region in terms of AMR, prevalence and genetic profiles. In general, there is a high prevalence of MDR *P. aeruginosa* seen in Egypt (75.6 %), with similarities between neighbouring countries, which might reflect comparable populations and antibiotic-prescribing cultures [13]. However, there is no literature available on the genomic diversity of *P. aeruginosa* isolates contributing to CAUTIs in Egypt. We therefore aimed to investigate the resistance and virulence gene profiles of *P. aeruginosa* contributing to CAUTIs by generating genome sequence data for isolates collected in an Egyptian hospital over a 3 month period, and compared their genotypic and phenotypic data with respect to AMR profiles and biofilm-forming abilities.

## Methods

### Recovery of isolates and ethical statement

Thirty-one *P. aeruginosa* isolates were recovered from urinary catheters between September and November 2021 by staff at the Urology and Nephrology Center, Mansoura University, Egypt during routine diagnostic procedures (Table 1). All isolates were associated with cases that had CAUTI as their primary diagnosis. We were informed that urine analysis had been performed on catheterized patients who presented with symptoms (mainly fever and dysuria). To collect a urine sample from patients with clinical signs/symptoms of a CAUTI, the urine had been aseptically aspirated from the urinary catheter and sent immediately to the hospital microbiology laboratory. Urine samples were examined under the microscope for white blood cells and processed using standard aseptic microbiological techniques. Urine samples were inoculated onto blood agar, cystine–lactose–electrolyte-deficient (CLED) agar and MacConkey agar plates, and incubated aerobically at 37 °C for up to 3 days. We were supplied with the cultures recovered on CLED agar, with only the date of isolation provided for samples in addition to confirmation of a CAUTI diagnosis; we were not provided with any patient data. Only a single colony type (with respect to colony morphology, colour, texture and size) was observed on each CLED agar plate, with the cultures assumed to represent mono-microbial infections. Confirmation of isolation of *P. aeruginosa* was further confirmed by inoculating colonies onto selective cetrimide agar in the microbiology laboratory of the Faculty of Pharmacy, Mansoura University.

**Table 1.** Summary information for the genomes generated from isolates described in this study (additional quality metrics can be found in Table S1)

Isolate	Isolated	Genome accession	Length (bp)	Contigs	N50	CDSs	ANI (%)*	ST
P1	23 September 2021	JAPWLO000000000	7 090 567	33	670 701	6518	99.28	244
P2	23 September 2021	JAPWLN000000000	7 561 602	176	218 286	6967	99.21	244
P3	23 September 2021	JAPWLM000000000	7 089 819	33	671 466	6519	99.25	244
P4	27 September 2021	JAPWLL000000000	6 567 076	29	731 473	5990	99.25	381
P5	27 September 2021	JAPWLK000000000	6 872 195	141	281 948	6358	98.71	773
P6	27 September 2021	JAPWLJ000000000	7 079 384	48	394 601	6519	99.27	244
P7	29 September 2021	JAPWLI000000000	6 595 040	42	716 476	6018	99.21	381
P8	29 September 2021	JAPWLH000000000	7 112 374	390	411 570	6551	98.74	773
P9†	5 October 2021	GCF_028595865.2	6 990 601	3	6 518 599	6498	99.29	3765
P10	5 October 2021	JAPWLF000000000	7 710 323	740	423 206	7002	99.20	381
P11	5 October 2021	JAPWLE000000000	6 585 784	41	457 535	6084	99.29	3765
P12	11 October 2021	JAPWLD000000000	6 589 324	58	656 238	6011	99.24	381
P13	11 October 2021	JAPWLC000000000	6 492 143	42	427 633	5924	99.26	1667
P14	11 October 2021	JAPWLB000000000	6 844 752	71	433 376	6327	98.73	773
P15	16 October 2021	JAPWLA000000000	6 577 280	114	456 538	6009	99.29	3765
P16	16 October 2021	JAPWKZ000000000	7 019 039	286	369 447	6394	98.78	357
P17	17 October 2021	JAPWKY000000000	6 845 094	62	327 266	6241	99.14	621
P18	17 October 2021	JAPWKX000000000	6 577 155	29	810 963	5993	99.24	381
P19†	20 October 2021	JAPWKW000000000	6 632 993	3	5 895 732	6036	99.22	381
P20	20 October 2021	JAPWKV000000000	6 835 420	70	316 419	6320	98.72	773
P22	20 October 2021	JAPWKU000000000	7 082 297	36	670 701	6522	99.28	244
P23†	26 October 2021	GCF_028595525.2	6 931 140	1	6 931 140	6287	99.14	621
P24†	26 October 2021	JAPWKS000000000	6 688 005	6	5 887 181	6109	99.23	381
P25	26 October 2021	JAPWKR000000000	6 642 761	33	457 730	6034	98.78	357
P26	26 October 2021	JAPWKQ000000000	6 827 640	99	307 141	6306	98.72	773
P27	27 October 2021	JAPWKP000000000	7 152 409	161	271 243	6601	98.69	773
P28	27 October 2021	JAPWKO000000000	6 410 783	55	322 863	5852	99.36	1430
P29	1 November 2021	JAPWKN000000000	6 757 213	145	400 482	6200	99.35	3765
P30	1 November 2021	JAPWKM000000000	6 836 605	79	411 378	6322	98.74	773
P31	1 November 2021	JAPWKL000000000	7 132 296	192	301 000	6570	98.70	357
P32	1 November 2021	JAPWKK000000000	6 665 983	94	383 436	6057	98.72	357

*Illumina-only assemblies compared (fastANI) with the genome of the type strain of *P. aeruginosa* (DSM 50071^T^; NCBI Genome Assembly GCF_012987025.1).

†Illumina plus Oxford Nanopore Technologies (ONT) Nanopore hybrid assembly.

The study of anonymized clinical isolates beyond the diagnostic requirement was approved by the Urology and Nephrology Center, Mansoura, Egypt. No other ethical approval was required for the use of the clinical isolates.

### Antimicrobial susceptibility testing

Antimicrobial susceptibility testing was performed using the disc diffusion test (DDT) on Mueller–Hinton agar (Oxoid Ltd, UK), with overnight cultures diluted to be equal to 0.5 McFarland standard (OD_600_=0.08–0.13) and spread (swabs) on the plates, followed by incubation at 37 °C for 18 h. Inhibition zone diameters were determined and recorded according to breakpoint tables of the European Committee on Antimicrobial Susceptibility Testing (EUCAST), version 12.0, 2022 (http://www.eucast.org/clinical_breakpoints/). The recommended EUCAST reference strain – *P. aeruginosa* ATCC 27853 – was used for quality control purposes in this study.

### Assay of biofilm formation

The assay was performed as described previously [14–16]. In brief, a single colony of each isolate was inoculated in 5 ml of tryptone soy broth (Oxoid Ltd) supplemented with 1 % (w/v) glucose (TSBG). Cultures were incubated aerobically for 24 h at 37 °C without shaking. The overnight cultures were diluted to 1 : 100 using TSBG and then aliquots (100 µl) of the diluted cultures were introduced into the wells of a 96-well plate. The plates were incubated aerobically for 24 h at 37 °C without shaking. Then, the spent medium was carefully removed from each well. The wells were washed three times with 200 µl sterile phosphate-buffered saline (pH 7.4; Oxoid Ltd) to remove any non-adherent planktonic cells. The adherent cells were fixed by heat treatment at 60 °C for 60 min to prevent widespread detachment of biofilms prior to dye staining. The adhered biofilms were then stained by addition of 1 % (w/v) crystal violet (150 µl per well) and the 96-well plate was left to incubate for 20 min. The excess stain was then carefully removed from the wells and discarded. The 96-well plate was carefully rinsed with distilled water three times and then the plate was inverted and left at room temperature until the wells were dry. The stained biofilms were solubilized by adding 33 % (v/v) glacial acetic acid (Sigma-Aldrich) to each well (150 µl per well). After solubilization of stained biofilms, the *A*_540_ was measured and recorded for all samples using a BioTek Cytation imaging reader spectrophotometer.

Uninoculated medium was used as a negative control in biofilm assays. Biological (*n*=3) and technical (*n*=4) replicates were performed for all isolates. *Salmonella enterica* serovar Enteritidis 27655S was used as a negative control in biofilm assays [17].

### DNA extraction and whole-genome sequencing (WGS)

For each isolate, a 500 µl aliquot of an overnight culture grown in nutrient broth (Oxoid Ltd) was used for DNA extraction using the Gentra Puregene Yeast/Bact. kit (Qiagen) according to the manufacturer’s instructions. Quality and quantity of the extracted DNA were checked by NanoDrop 2000/2000 c (Thermo Fisher Scientific).

Illumina sequencing (Nextera XT Library Prep kit; HiSeq/NovaSeq; 2×250 bp paired-end reads; minimum 30× coverage, mean value after trimming and filtering of reads) was performed by microbesNG (Birmingham, UK) as described previously [18]. In brief, reads were adapter-trimmed to a minimum length of 36 nt using Trimmomatic 0.30 [19] with a sliding window quality cut-off of Q15. *De novo*-assembled genomes (SPAdes v3.7 [20]) were returned to us by microbesNG.

Genomic DNA for four isolates (P9, P19, P23 and P24) was further sequenced to obtain long-read sequences using an Oxford Nanopore Technologies (ONT) MinION. The ligation sequencing kit SQK-LSK109 and native barcoding kit EXP-NBD104 were used for Nanopore library preparation. Libraries were loaded onto a MinION R9.4.1 flow cell and run for 48 h. Fast5 files were basecalled using the SUP (super high accuracy) model of Guppy v6.4.2 and subsequently demultiplexed. Porechop (https://github.com/rrwick/Porechop) was used to trim end and middle adapter sequences and reads shorter than 1 kbp were discarded using Filtlong v0.2.1 (https://github.com/rrwick/Filtlong). Nanopore reads were *de novo* assembled using Flye v2.9.1 [21]. Closed genomes were manually reoriented to begin with *dnaA*, prior to polishing with both Nanopore and Illumina reads. Assembled sequences were polished with Nanopore reads using four iterations of Racon v1.5.0 [22], followed by Medaka v1.7.2 and Homopolish v0.3.4 [23]. Resulting sequences were then polished with Illumina reads using Polypolish v0.5.0 [24], POLCA from the MaSuRCA v4.0.9 package [25] and Nextpolish v1.4.1 [26].

### Bioinformatic analyses

Contigs with <500 bp were filtered from draft genomes using reformat.sh of BBmap 38.97 [27]. CheckM v1.2.1 was used to assess genome assembly quality with respect to percentage completeness and contamination [28]. The identity of isolates as *P. aeruginosa* was confirmed by average nucleotide identity analysis (ANI) (fastANI v1.3.3) [29] against the genome of the type strain of the species (DSM 50071^T^, NCBI Genome Assembly GCF_012987025.1), as is routine practice when determining taxonomic affiliations of newly isolated strains based on genomic data [30]. Bakta v1.5.1 (database v4.0) was used for annotating genes within genomes [31]. The Bakta-annotated whole-genome sequence data are available from figshare in GenBank format. The Virulence Factor Database (VFDB) [32] was used to predict virulence genes encoded within genomes. The multilocus sequence type (MLST) of each isolate was determined using the MLST schema for *P. aeruginosa* at PubMLST (http://pubmlst.org/paeruginosa) [33, 34]. PubMLST summary data were downloaded for 8435 isolates on 16 December 2022. Antimicrobial resistance markers were identified using the Resistance Gene Identifier (RGI) v6.0.0 tool of the Comprehensive Antibiotic Resistance Database (CARD) v3.2.5 [35]. Only resistance genes that showed a perfect or strict match with coverage for a given gene in the database are reported in this study. Phylogenetic analysis of genomic data was carried out using PhyloPhlAn 3.0 (--diversity low -f supermatrix_aa.cfg) [36] with 245 *Pseudomonas* reference sequences downloaded from the Genome Taxonomy Database, release 07-RS207 (Supplementary Material: gtdb-search.csv) [37].

A blastn search (--outfmt 6) was made using the megaplasmid pBT2436-like core gene sequences (*repA*, *parA*, *virB4*) described by Cazares *et al*. [38] against the contigs of our newly generated short-read genome sequence data. In addition, the reads from our short-read sequence data were trimmed to ≥70 nt each using cutadapt v4.1 [39] and then mapped using BWA-MEM v.0.7.17-r1188 [40] against the reference megaplasmid sequences shown in Table 2. The presence of pBT2436-like megaplasmids in our genomes was assessed based on the percentage of reads mapped to the reference genomes of Cazares *et al*. [38] as extracted from the alignment files with samtools v.1.16.1 [41]. plaSquid was used to further characterize the plasmids [42].

**Table 2.** pBT2436-like megaplasmid reference sequences included in this study

Plasmid	Species and strain	Size (bp)	No. of predicted genes^*^	Country	Source	GenBank accession	Reference(s)
pBT2436	* P. aeruginosa* 2436	422 811	537	Thailand	Respiratory infection	CP039989	[38]
pBT2101	* P. aeruginosa* 2101	439 744	556	Thailand	Respiratory infection	CP039991	[38]
unnamed2	* P. aeruginosa* AR_0356	438 531	557	Unknown	Unknown	CP027170	[38]
unnamed2	* P. aeruginosa* AR439	437 392	549	Unknown	Unknown	CP029096	[38]
unnamed3	* P. aeruginosa* AR441	438 529	560	Unknown	Unknown	CP029094	[38]
pJB37	* P. aeruginosa* FFUP_PS_37	464 804	597	Portugal	Respiratory infection	KY494864	[38, 104]
pBM413	* P. aeruginosa* PA121617	423 017	537	PR China	Respiratory infection	CP016215	[38, 105]
pOZ176	* P. aeruginosa* PA96	500 839	621	PR China	Respiratory infection	KC543497	[38, 106]
p12939-OXA	* P. aeruginosa* (unknown)	496 436	607	PR China	Unknown	MF344569	[38]
p727-IMP	* P. aeruginosa* (unknown)	430 173	534	PR China	Unknown	MF344568	[38]
pA681-IMP	* P. aeruginosa* (unknown)	397 519	486	PR China	Unknown	MF344570	[38]
pR31014-IMP	* P. aeruginosa* (unknown)	374 000	456	PR China	Unknown	MF344571	[38]
pRBL16	* P. citronellolis* SJTE-3	370 338	486	PR China	Wastewater sludge	CP015879	[38, 107]
p1	* P. koreensis* P19E3	467 568	598	Switzerland	*Origanum majorana*	CP027478	[38, 108]
pSY153-MDR	* P. putida* SY153	468 170	579	PR China	Urinary tract infection	KY883660	[38, 109]

*Predicted in this study using Bakta.

Complete *Pseudomonas* plasmid sequences were downloaded from NCBI Genome on 19 December 2022 (Supplementary Material: plasmids.csv), and filtered to retain genomes >200 000 bp. These sequences were subject to blastn (--outfmt 6) searches against the pBT2436 sequences for *repA*, *parA* and *virB4* as described above. Those plasmid sequences returning single-copy hits for the three genes were subject to further analyses as follows.

For comparative analyses, the megaplasmid sequences were annotated using Bakta as described above for the *Pseudomonas* genome sequences. The Bakta-annotated plasmid sequence data are available from figshare in GenBank format. FastANI v1.33 [29] was used to determine how similar the sequences of the newly identified megaplasmids were to those of pBT2436 and other reference genomes (Table 2); visualization of the conserved regions between pairs of plasmid sequences was achieved using the --visualize option of FastANI and the R script available at https://github.com/ParBLiSS/FastANI. The protein sequences predicted to be encoded by all the plasmids were concatenated, sorted by length (longest to shortest) using vsearch v2.15.2_linux_x86_64 [43] and clustered using MMseqs2 v13.45111 [44] (80 % identity, 80 % coverage). Those core sequences found in MMseqs2 clusters in single copies in all plasmids [38] were concatenated and used to generate a sequence alignment (MAFFT v7.490, BLOSUM 62; Geneious Prime v2023.0.1) from which a GAMMA BLOSUM62 substitution model maximum-likelihood tree (RAxML 8.2.11 [45]; parameters selected to generate best-scoring maximum-likelihood tree, 100 bootstraps; Geneious Prime v2023.0.1) was generated. The bespoke R script associated with processing of the sequence data along with all output files are provided as Supplementary Material on figshare.

### Characterization of phenotypic and genomic concordance/discordance

For easier description and discussion of phenotypic and genomic results, we grouped the ‘susceptible, standard dosing regimen’ (S) and ‘susceptible, increased exposure’ (I) categories under the term ‘susceptible’, as currently recommended by EUCAST. WGS data were compared with DDT data for 31 *Pseudomonas* isolates against 10 antimicrobials (*n*=310 combinations). For each combination, concordance was considered positive if (a) WGS data were predicted to encode AMR genes and the isolate had a phenotypic resistant profile (WGS-R/DDT-R) or (b) WGS data were not predicted to encode AMR genes and the isolate had a phenotypic susceptible profile (WGS-S/DDT-S) as described previously [46, 47]. Discordance was considered positive in the case of major or very major errors. Major errors (WGS-R/DDT-S) are defined as a resistant genotype and a susceptible phenotype. Very major errors (WGS-S/DDT-R) are defined as a susceptible genotype and a resistant phenotype. WGS results were classified as ‘resistant’ when one or several AMR genes were identified by CARD and allocated as the mechanism of AMR to that antimicrobial, and as ‘susceptible’ when no AMR gene was found.

## Results

### Genome characterization

The draft genomes assembled from short-read data consisted of between 29 and 740 contigs; the hybrid-assembled genomes consisted of between 1 and 6 contigs. All were of high quality (i.e. completeness >90 %, contamination <5 % [48]). Between 4 and 12 rRNA genes were predicted to be encoded within the genomes. Only 1 (P27, 481 nt) of the 31 genomes did not encode at least 1 copy of the 16S rRNA gene ≥1000 nt in length; 24 of the genomes encoded complete (1536 nt) 16S rRNA genes, with P9 and P19 both encoding 4 copies of the 16S rRNA gene (Table S1). The mean number of coding sequences predicted to be encoded within the genomes was 6295±283. Genomes had a mean G+C content of 66 %. The tRNA copy number for the isolates ranged from 59 to 70. All isolates were confirmed to be *P. aeruginosa* by ANI analysis against the genome of the type strain of *P. aeruginosa* (>95–96 % ANI [30]), with additional support provided by phylogenetic analysis (Fig. S1). The general features of the isolates’ genomes are provided in Tables 1 and S1.

### Genotypic and phenotypic AMR profiles

**Fig. 1.**
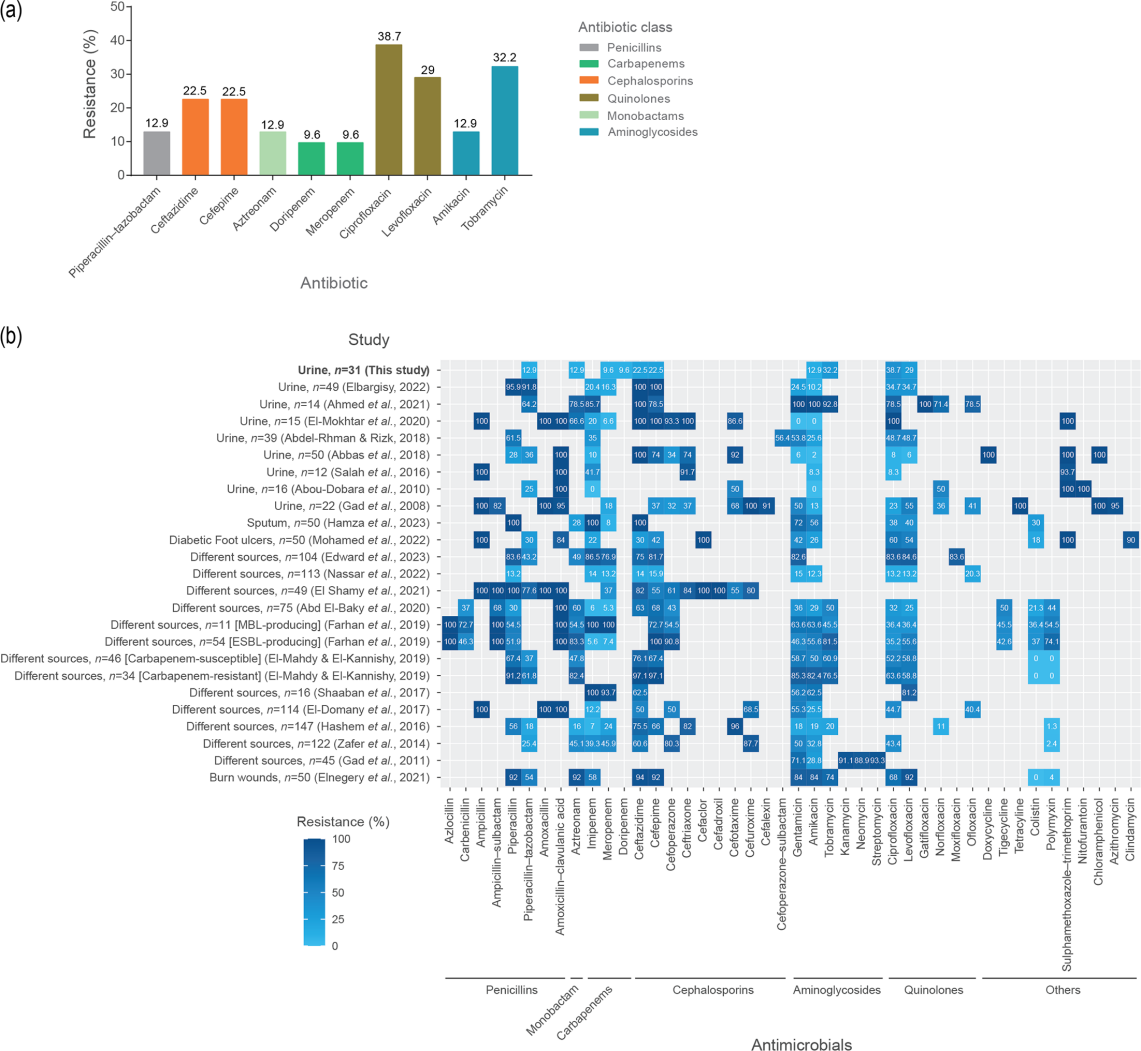
Classes of antimicrobials to which the 31 *P. aeruginosa* isolates recovered from CAUTIs were resistant, and comparison of results with previous studies from Egypt. (**a**) AMR susceptibility testing was performed according to EUCAST guidelines. The figure depicts the proportion (%) of isolates that were resistant to each antibiotic. (**b**) Previous reports for AMR found in *Pseudomonas* isolated from different sources in Egyptian clinical settings. Data are taken from a range of the literature [84, 86, 110–128, 129]. ESBL, extended-spectrum β-lactamase; MBL, metallo-β-lactamase.

The AMR profiles of the 31 *P *. *aeruginosa* isolates were determined according to EUCAST guidelines. A summary of the classes of antimicrobials the isolates were resistant to is provided in Fig. 1a. The isolates were highly resistant to quinolones [ciprofloxacin (*n*=12/31, 38.7 %) and levofloxacin (*n*=9/31, 29 %)] followed by tobramycin (*n*=10/31, 32.5 %) and cephalosporins (*n*=7/31, 22.5 %). Six (P5, P18, P20, P26, P28, P30) of the 31 isolates (19.3 %) were MDR (i.e. resistant to ≥3 antimicrobials from 3 different antibiotic classes) (Table 3). Previous reports from Egypt showed a mean percentage of AMR for isolates from urine of 13 % for meropenem, 19 % for amikacin, 36 % for levofloxacin and 43 % for ciprofloxacin (Fig. 1b). However, the mean percentage was higher (50–100 %) for aztreonam, piperacillin/tazobactam, ceftazidime, cefepime and tobramycin.

**Table 3.** Overview for resistance genes of MDR isolates of *P. aeruginosa *. All isolates were predicted to encode the aminoglycoside-modifying enzyme *APH(3′)-IIb*

Isolate	β-lactamases	Resistance to fluoroquinolones	Others	Efflux pump systems	Phenotypic resistance profile*
P5	*OXA-395* *PDC-16*	*gyrA qnrVC1*	*fosA catB7* *sul1*	MexAB-OprM MexCD-OprJ MexEF-OprN MexHI-OpmD MexPQ-OpmE	AK ATM CIP FEP LEV TOB
P18	*OXA-50* *PDC-14*	–	*fosA catB7*	MexAB-OprM MexCD-OprJ MexEF-OprN MexHI-OpmD MexPQ-OpmE	ATM CIP TOB CAZ FEP
P20	*NDM-1* *OXA-395* *PDC-16*	*gyrA qnrVC1*	*fosA catB7* *cmlA9* *sul1* *tet(D*)	MexAB-OprM MexCD-OprJ MexEF-OprN MexHI-OpmD MexPQ-OpmE	AK CAZ CIP DOR FEP LEV MEM TOB TZP
P26	*NDM-1* *OXA-395* *PDC-16*	*gyrA qnrVC1*	*fosA catB7* *cmlA9* *sul1* *tet(D*)	MexAB-OprM MexCD-OprJ MexEF-OprN MexHI-OpmD MexPQ-OpmE	AK CAZ CIP DOR FEP LEV MEM TOB TZP
P28	*OXA-903* *PDC-3*	–	*fosA catB7*	MexAB-OprM MexCD-OprJ MexEF-OprN MexHI-OpmD MexPQ-OpmE	ATM CAZ CIP FEP TZP
P30	*NDM-1* *OXA-395* *PDC-16*	*gyrA qnrVC1*	*fosA catB7* *cmlA9* *sul1* *tet(D*)	MexAB-OprM MexCD-OprJ MexEF-OprN MexHI-OpmD MexPQ-OpmE	AK CAZ CIP DOR FEP LEV MEM TOB TZP

*AK, amikacin; ATM, aztreonam; CAZ, ceftazidime; CIP, ciprofloxacin; DOR, doripenem; FEB, cefepime; LEV, levofloxacin; MEM, meropenem; TOB, tobramycin; TZP, piperacillin–tazobactam.

**Fig. 2.**
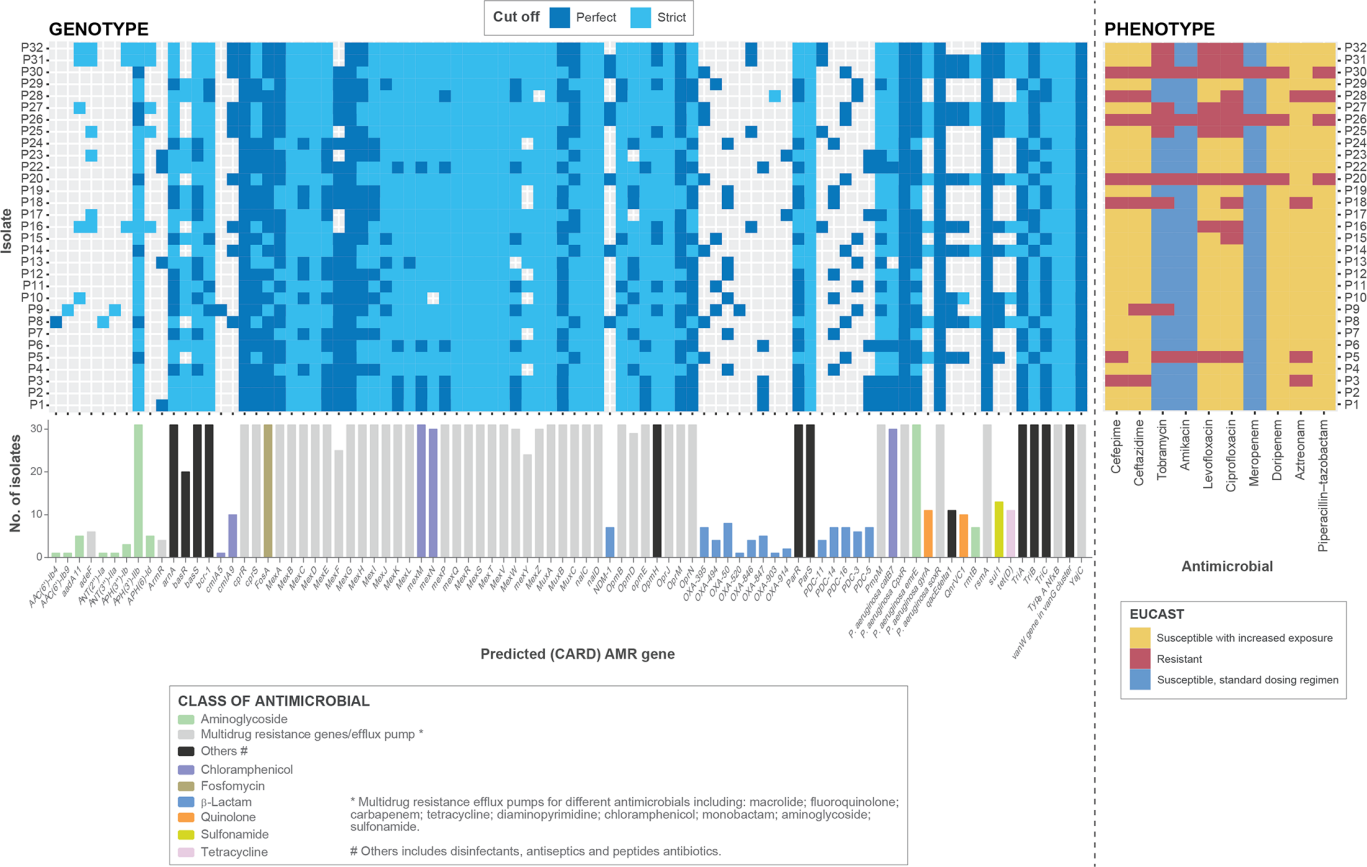
AMR genes predicted to be encoded within the genomes of the 31 isolates compared with their AMR phenotypic profiles (determined according to EUCAST guidelines). Resistomes were characterized using the RGI tool of CARD for perfect and strict hits. Strict CARD match, not identical but the bit score of the matched sequence is greater than the curated blastN bit score cut-off; perfect CARD match, 100 % identical to the reference sequence along its entire length. The bar graphs under the genotypic data show the number of genomes encoding each predicted AMR gene.

Through genotypic analysis using RGI/CARD, a total of 88 antibiotic resistance genes were predicted to be encoded by the 31 isolates (726 perfect hits and 1182 strict hits), including genes conferring resistance to β-lactams, aminoglycosides, fluoroquinolones, macrolides and tetracyclines through different mechanisms, such as antibiotic efflux and antibiotic target alteration (*n*=175), antibiotic inactivation (*n*=179), antibiotic efflux (*n*=1389), antibiotic target alteration (*n*=80), reduced permeability to antibiotics (*n*=62), antibiotic target protection (*n*=10) and antibiotic target replacement (*n*=13). RGI/CARD results for the *P. aeruginosa* isolates are summarized in Fig. 2 and compared with the phenotypic data.

In terms of comparing genotypic with phenotypic profiles for the MDR isolates, P5, P18, P20, P26, P28 and P30 were predicted to encode an aminoglycoside-modifying enzyme [APH(3’)-Ilb] and five efflux pump systems (MexAB-OprM, MexCD-OprJ, MexEF-OprN, MexHI-OpmD and MexPQ-OpmE), while 4/6 and 5/6 of the MDR isolates were phenotypically resistant to the aminoglycosides amikacin and tobramycin, respectively. The genomes of isolates P20, P26 and P30 were also predicted to encode the β-lactamases *NDM-1*, *OXA-395* and *PCD-16*; isolate P5 encoded *OXA-395* and *PDC-16*; isolate P18’s genome was predicted to encode *OXA-50* and *PDC-14*; isolate P28 was predicted to encode *OXA-903* and *PDC-3*. Phenotypically, 5/6 and 6/6 of the MDR isolates were resistant to ceftazidime and cefepime, respectively. Genes conferring resistance to quinolones (*gyrA* and *qnrVC1*) were predicted to be harboured by isolates P5, P20, P26 and P30 (Table 3).

There were many additional resistance determinants predicted to be encoded within the genomes of the susceptible isolates with increased exposure (I): aminoglycoside-modifying enzymes *AAC(6′)-Ib4*, *AAC(6′)-Ib9*, *aadA11*, *ANT(2″)-Ia*, *ANT(3″)-IIa*, *APH(3″)-Ib*, *APH(3′)-IIb*, *APH(6)-Id* and the β-lactamases *OXA-50*, *OXA-395*, *OXA-494*, *OXA-520*, *OXA-846*, *OXA-847*, *OXA-903*, *OXA-914*, *PDC-3*, *PDC-5*, *PDC-11*, *PDC-14* and *PDC-16* (Fig. 2).

Comparison of our WGS data and DDT results (with respect to predicted AMR genes and actual resistance phenotypes) yielded a concordance of 31 %, with discordant results (69 %) mainly due to phenotypically susceptible isolates predicted to encode AMR determinants in their genomes (e.g. isolate P20 concordant for resistance to piperacillin–tazobactam, but discordant for aztreonam; Table S2). However, the discordant cases were not equally distributed. In 68.1 % of discordant cases, one or several AMR genes were predicted in the genome but the isolate was phenotypically susceptible (major errors, WGS-R/DDT-S; e.g. isolate P1 for the cephalosporins ceftazidime and cefepime). The remaining 0.9 % discordances were phenotypically resistant isolates in which no genetic determinants of AMR were predicted (very major errors, WGS-S/DDT-R; e.g. isolate P18 for the fluoroquinolone ciprofloxacin) (Table S2).

### Biofilm formation

**Fig. 3.**
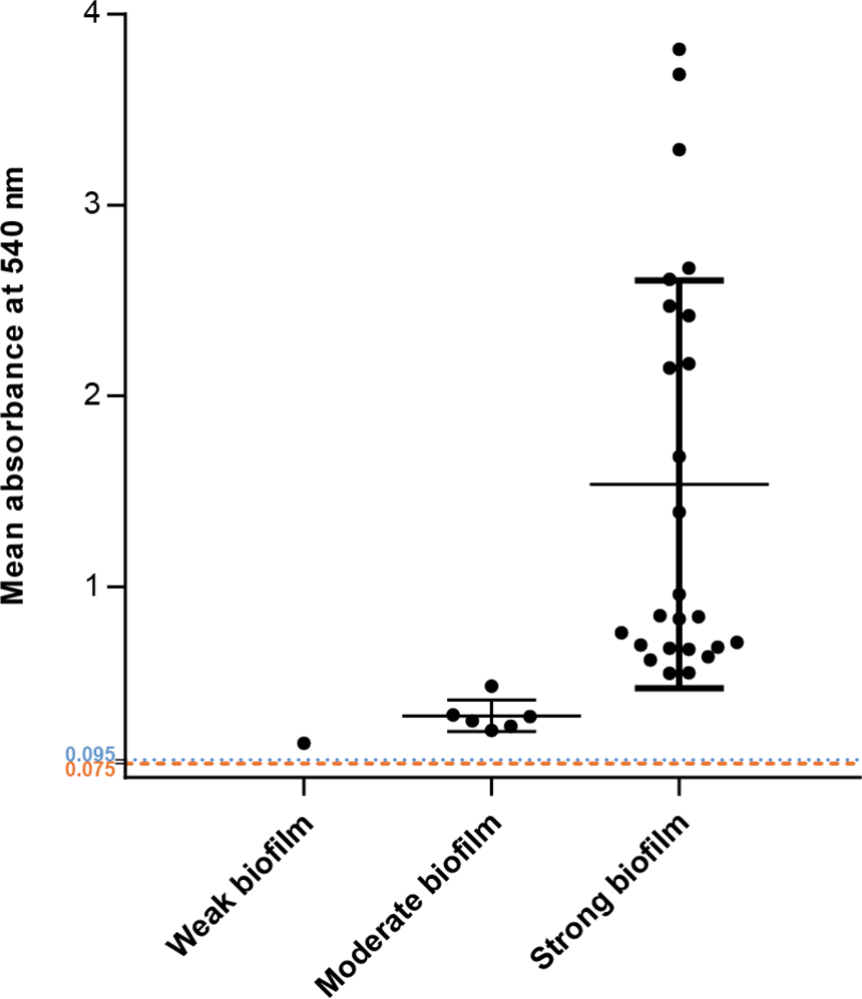
Classification of *P. aeruginosa* isolates according to their capacity to produce biofilm in TSBG. Data for each isolate are represented as the mean of four technical replicates (three biological replicates each). The blue dashed line (0.095) represents *Salmonella enterica* serovar Enteritidis 27655S, while the orange dashed line (0.075) represents the uninoculated medium. The mean and its standard deviation are represented for each biofilm formation category.

The biofilm-forming abilities of the 31 isolates were tested and compared with a known biofilm-negative control (* Salmonella enterica* serovar Enteritidis 27655S). *P. aeruginosa* isolates tended to form strong biofilms, with the isolates’ biofilm-forming ablilities classified as follows: non-biofilm producer (no change in *A*_540_ over the medium control=0.075); weak biofilm producer (up to a twofold change over the control); moderate biofilm producer (up to fourfold change over the control); strong biofilm producer (greater than fourfold change over the control) [16]. The majority (77.4 %) of the isolates were strong biofilm-producers (P1, P3, P4, P5, P8, P9, P11, P12, P13, P14, P15, P17, P18, P19, P20, P22, P23, P25, P26, P27, P28, P30, P31, P32), 19.3 % were moderate (P2, P6, P7, P10, P16, P24) and 3.2 % were weak (P29) (Fig. 3).

### Virulence factors associated with adherence and secretion systems

The investigation of virulence factors using VFDB predicted that isolates encode various virulence genes, ranging from 196 to 210 in number per isolate. Genes with no known functionality – ‘undetermined’ in the VFDB database – were excluded from further analysis. The major functional attributes of the known virulence factor genes detected in genomes were adherence (37.2 % abundance) and secretion systems (22 % abundance). All virulence genes detected by VFDB analysis are mentioned in Table S3.

### MLST revealed multiple major clonal complexes

The clonal diversity among the 31 *P. aeruginosa* isolates showed 8 different sequence types (STs): ST244, ST357, ST381, ST621, ST773, ST1430, ST1667 and ST3765 (Table 1). There were no relevant data in the PubMLST database regarding STs of *P. aeruginosa* in Egypt, although it is in the centre of the MENA region. We therefore compared the STs of the PubMLST database with those of our isolates, with respect to other countries and sources of infection (Table 4). The STs of *P. aeruginosa* in our study matched those of isolates detected outside the MENA region. PubMLST reported data for 107 ST244 isolates, 35 ST357 isolates, 47 ST381 isolates, 4 ST621 isolates, 10 ST773 isolates and 1 isolate each of ST1430, ST1667 and ST3765 across a range of non-MENA countries. Reported isolates of the MENA region had unique STs. The previous reported STs relevant to the MENA region are shown in Table 5. The previous STs associated with UTIs are ST244 [Poland (4), Australia (1), Brazil (2)], ST357 [Poland (2)] and ST381 [Malaysia (1)].

**Table 4.** Summary of STs found in PubMLST database that matched those detected in this study

ST in current study	Source of isolation (*n* isolates)	Relevant countries (*n* isolates)
ST244	Blood (14) Bronchial lavage (3) Other (19) Soft tissue infection (7) Sputum (3) **Urinary tract infection (7**)* Hospital effluent (3) Water (2) Soil (1)	Australia (10) Brazil (12) Central African Republic (3) PR China (1) France (10) Ghana (1) Ivory Coast (2) Nigeria (2) Poland (14) Russia (3) Spain (7) UK (1) Unknown (41)
ST357	Bronchial lavage (6) Water (1) Other (5) Soft tissue infection (2) Sputum (2) **Urinary tract infection (2**)	Brazil (2) France (1) Ghana (1) Malaysia (2) Nigeria (1) Peru (4) Poland (5) Senegal (1) Singapore (1) Unknown (17)
ST381	Blood (6) Other (11) Soft tissue infection (1) Sputum (2) Water (2) Hospital effluent (1) **Urinary tract infection (1**)	Australia (7) Brazil (1) France (4) Ivory Coast (4) Malaysia (2) Poland (3) Russia (3) Spain (1) Unknown (22)
ST621	Unknown	Austria (1) Unknown (3)
ST773	Soft tissue infection (3) Other (1) Sputum (1) Blood (1)	Bangladesh (1) Central African Republic (1) PR China (1) Ghana (3) Russia (1) Unknown (3)
ST1430	Unknown	Unknown (1)
ST1667	Unknown	PR China (1)
ST3765	Sputum (1)	Russia (1)

*Bold text, associated with UTI.

**Table 5.** Summary for relevant STs found in PubMLST of *P. aeruginosa* in MENA region

Country	Source of infection (*n* isolates)	Relevant ST(s)
Algeria	Blood (1)	674
	Other (2)	3349, 3350
Iran	Soft tissue infection (2)	967, 972
	Sputum (5)	3118, 3119, 3377, 3381, 3382, 3450
	**Urinary tract infection (5**)*	**970, 3376, 3378, 3379, 3380**
Iraq	Bronchial lavage (1)	2209
	Other (2)	2203, 2208
	Soft tissue infection (9)	2196, 2197, 2198, 2199, 2200, 2201, 2202, 2205, 2206
	Sputum (2)	2204, 2207
	**Urinary tract infection (3**)	**2195, 2210, 3352**
Kuwait	Unknown (1)	3842
Lebanon	Bronchial lavage (1)	1702
	Other (5)	1701, 1759, 1760, 1761, 1762
	**Urinary tract infection (3**)	**1699, 1700, 3425**
	Unknown (1)	3985
Libya	Sputum (5)	1924, 1925, 1926, 1927, 1928
Palestine	Soft tissue infection (3)	1562, 1563, 1564
Saudi Arabia	Sputum (2)	3728, 3729
	**Urinary tract infection (1**)	**3730**
	Unknown (12)	2010, 2012, 2013, 3710, 3711, 3712, 3713, 3714, 3715, 3716, 3717, 3718
Sudan	Blood (2)	3900
	**Urinary tract infection (3**)	**3898, 3899, 3901**
Tunisia	Other (11)	2042, 2043, 2537, 2538, 3385, 3386, 3968, 3969, 3970
	Sputum (1)	3762
	Water (1)	2539
Turkey	Blood (2)	2529, 2531
	Bronchial lavage (1)	2532
	Other (1)	2034
	Soft tissue infection (15)	2513, 2514, 2515, 2516, 2516, 2517, 2518, 2519, 2520, 2521, 2522, 2523, 2525, 2526, 2527
	**Urinary tract infection (2**)	**2528, 2530**
United Arab Emirates	Sputum (1)	2011

*Bold text, associated with UTI.

### Megaplasmid identification

**Fig. 4.**
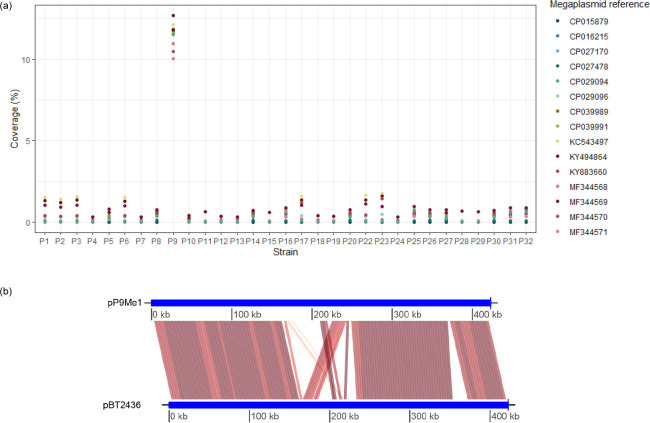
Detection and characterization of a pBT2436-like megaplasmid in the genome of *P. aeruginosa* P9. (**a**) Proportion of Illumina sequence reads generated for *P. aeruginosa* isolates recovered in Egypt that map to pBT2436-like megaplasmid reference sequences. (**b**) Visualization of the conserved regions between the sequences of the megaplasmids pP9Me1 and pBT2436 as determined using FastANI, with *repA* set as the start gene for both plasmid sequences.

Visual inspection of Bandage maps (not shown) generated for our short-read draft genome assemblies suggested that isolate P9 encoded a circular megaplasmid of >400 000 bp. The *repA*, *parA* and *virB4* sequences of megaplasmid pBT2436 were extracted from its sequence (accession CP039989) using the PCR primer sequences of Cazares *et al*. [38]. These were used in a blastn search of the draft genomes for all our *P. aeruginosa* isolates. P9 returned hits, sharing 97.1, 99.4 and 100 % similarity with the *repA*, *parA* and *virB4* nucleotide sequences, respectively. Confirmation of isolate P9 encoding a circular pBT2436-like megaplasmid was achieved by mapping the reads of all isolates against the genomes of the reference genomes [38] listed in Table 2. Between 10.01 and 12.68 % of the Illumina reads of isolate P9 mapped to the pBT2436-like megaplasmid reference genomes (Fig. 4a). No other isolate had more than 1.8 % of its reads map to any of the reference megaplasmid sequences.

Consequently, a MinION/Illumina hybrid assembly was generated for P9 (Table 1). The genome comprised a complete, circular chromosome (6 518 599 bp) and two complete, circular plasmids (pP9Me1, 422 938 bp; pP9Me2, 49 064 bp). The chromosome was predicted to encode 5950 CDSs. Neither plasmid matched sequences in PlasmidMLST. The megaplasmid pP9Me1 was assigned to PTU-Pse13 (score 1.000) by COPLA [49]. pP9Me2 could not be assigned to a plasmid taxonomy unit using this tool. No mobility group, replication initiator protein domain or replicon type could be assigned to pP9Me1 or pP9Me2 by plaSquid. However, Bakta did identify a replication initiation protein (RepA) in pP9Me2’s sequence that shared homology with UniRef90_A0A218MAR0, a HK97 gp10 family phage protein of *P. aeruginosa *.

The megaplasmid pP9Me1 was predicted to encode 538 CDSs, including the virulence genes (VFDB) *pilD* (type IV pili biosynthesis), *chpA* and *pilG* (type IV pili twitching motility-related proteins) and *csrA* (carbon storage regulator A), and the AMR genes *sul1*, *qacEdelta1*, *OXA-520*, *cmlA5* (CARD perfect matches) plus *ANT(3″)-Iia* and *AAC(6′)-Ib9* (CARD strict matches). Its sequence shared high similarity with that of pBT2436; a progressiveMauve alignment (not shown) of the sequences of pBT2436 and pM9Me1 showed them to share 163 628 identical sites (97 % pairwise identity), and they shared an ANI (fastANI) of 98.5 % (Fig. 4b).

Plasmid pP9Me2 was predicted to encode 68 CDSs; it did not encode any AMR- or virulence-associated genes based on CARD and VFDB searches. Based on an NCBI blastn analysis, its sequence shared high similarity with the circular and complete (50 754 bp; GenBank accession CP081288.1) *P. aeruginosa* plasmid pF092021-1 (93 % query coverage, 98.7 % identity; Fig. S2). A progressive Mauve alignment of the sequences showed pP9Me2 and pF092021-1 to share 44 425 identical sites (81.1. % pairwise identity) (Fig. S3); ANI could not be determined for these plasmid sequences.

**Fig. 5.**
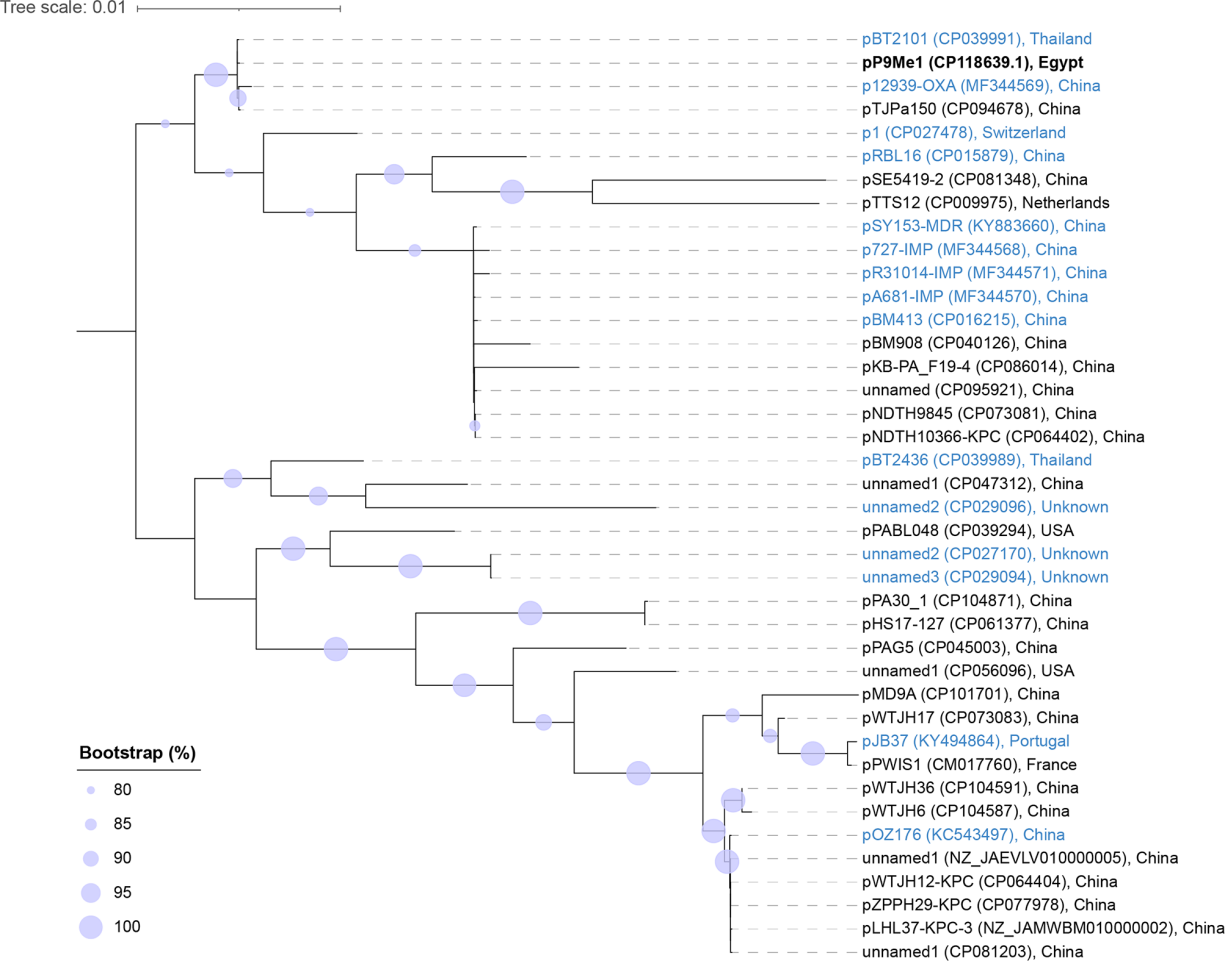
Phylogenetic (maximum-likelihood) tree showing the relationships of pP9Me1 and other pBT2436-like megaplasmids. The tree, rooted at the midpoint, was built from a multiple-sequence alignment of 55 243 aa, comprising the sequences of 217/261 core proteins described by Cazares *et al*. [38]. Plasmids shown in blue were defined as pBT2436-like by Cazares *et al*. [38], while those in black were identified as pBT2436-like in the current study. Scale bar, average number of amino acid substitutions per position. The tree shown represents the best-scoring maximum-likelihood tree as determined using RAxML (parameters -f a -x 1). Bootstrap values determined based on 100 replications.

In their original study, Cazares *et al*. [38] identified 15 pBT2436-like megaplasmids (Table 2). blastn searches (Supplementary Material: blastn_hits_plasmids.xlsx) of the pBT2436 *repA*, *parA* and *virB4* sequences against all complete *Pseudomonas* plasmid sequences >200 000 bp from NCBI Genome identified a further 24 potential pBT2436-like megaplasmids encoding only 1 copy each of the 3 pBT2436-like sequences (Table 6). FastANI analysis showed that the sequences of these plasmids shared between 95.9 and 100 % ANI with one another, pP9Me1 and the 15 reference sequences (Fig. S4). Consequently, the protein sequences predicted to be encoded by the 40 megaplasmids were clustered, to identify single-copy proteins that shared 80 % identity and 80 % coverage with the core sequences of pBT2436 [38]. Of the 261 core sequences described for pBT2436, 217 were included in our analysis. We found an alignment (55 243 aa) of these concatenated sequences to share between 97.4 and 100 % identity, with the sequences of plasmids pWTJH12-KPC (CP064404) and pZPPH29-KPC (CP077978) identical to one another (they were from isolates recovered in the same hospital) [50]. Phylogenetic analysis (maximum likelihood) showed that pP9Me1 clustered with pBT2436-like plasmids identified previously [38] [especially two plasmids from PR China (p12939-OXA, pTJPa150) and one from Thailand (pBT2101); 100 % bootstrap support], but in a clade distinct from that with pBT2436 (Fig. 5).

**Table 6.** New *Pseudomonas* pBT2436-like megaplasmids identified in this study

Plasmid	Species and strain	Size (bp)	CDSs	Country	Source	Accession	blast similarity (%)			Reference
							***parA***	***repA***	***virB4***	
pP9Me1	* P. aeruginosa* P9	422 938	538	Egypt	CAUTI	CP118639.1	99.4	97.1	100.0	This study
pPWIS1	* P. aeruginosa* TC4411	419 683	529	France	Urine	CM017760.1	99.8	97.1	99.7	–
pTTS12	* P. putida* S12	583 900	669	Netherlands	Soil	CP009975.1	99.8	99.7	99.7	[130]
pPABL048	* P. aeruginosa* PABL048	414 954	521	USA	Blood (bacteraemia)	CP039294.1	99.6	97.1	99.0	[131]
pBM908	* P. aeruginosa* PA298	395 774	513	PR China	Human gut	CP040126.1	99.4	97.1	99.7	–
pPAG5	* P. aeruginosa* PAG5	513 322	653	PR China	Urine	CP045003.1	99.4	97.1	99.7	105
unnamed1	* P. putida* YC-AE1	504 084	623	PR China	Soil	CP047312.1	100.0	99.7	99.6	[132]
unnamed1	* P. aeruginosa* PABCH09	510 959	635	USA	Endotracheal tube	CP056096.1	99.8	97.1	99.0	[133]
pHS17-127	* P. aeruginosa* HS17-127	486 963	617	PR China	Urine	CP061377.1	99.4	97.1	99.7	134
pNDTH10366-KPC	* P. aeruginosa* NDTH10366	392 244	509	PR China	Human	CP064402.1	99.4	97.1	99.7	[135]
pWTJH12-KPC	* P. aeruginosa* WTJH12	396 963	515	PR China	Human	CP064404.1	99.8	97.1	99.7	[135]
pNDTH9845	* P. aeruginosa* NDTH9845	463 517	587	PR China	Human	CP073081.1	99.4	97.1	99.7	50
pWTJH17	* P. aeruginosa* WTJH17	436 486	548	PR China	Human	CP073083.1	99.8	97.1	99.7	50
pZPPH29-KPC	* P. aeruginosa* ZPPH29	397 554	511	PR China	Human	CP077978.1	99.8	97.1	99.7	[135]
unnamed1	* P. aeruginosa* P9W	475 028	605	PR China	Burn wound	CP081203.1	99.8	97.1	99.7	[136]
pSE5419-2	* P. aeruginosa* SE5419	478 017	595	PR China	Unknown	CP081348.1	99.8	99.7	99.7	[137]
pKB-PA_F19-4	* P. aeruginosa* KB-PA_F19	412 187	528	PR China	Burn wound	CP086014.1	99.4	97.1	99.7	[138]
pTJPa150	* P. aeruginosa* Pa150	436 716	544	PR China	Tissue (diabetic foot)	CP094678.1	99.4	97.1	100.0	[139]
unnamed	* P. aeruginosa* AR19640	495 621	599	PR China	Rectal swab	CP095921.1	99.4	97.1	99.7	[140]
pMD9A	* P. asiatica* MD9	455 169	574	PR China	Water (poultry farm)	CP101701.1	99.8	99.7	99.7	–
pWTJH6	* P. aeruginosa* WTJH6	426 499	529	PR China	Human	CP104587.1	99.8	97.1	99.7	–
pWTJH36	* P. aeruginosa* WTJH36	462 066	576	PR China	Human	CP104591.1	99.8	97.1	99.7	–
pPA30_1	* P. aeruginosa* PA30	453 250	565	PR China	CAUTI	CP104871.1	99.4	97.1	99.7	–
unnamed1	* P. aeruginosa* PA1120	437 632	567	PR China	Sputum	NZ_JAEVLV010000005.1	99.8	97.1	99.7	[141]
pLHL37-KPC-3	* P. aeruginosa* LHL-37	394 987	511	PR China	Sputum	NZ_JAMWBM010000002.1	99.8	97.1	99.7	–

## Discussion

Genomes of *P. aeruginosa* are complex and highly variable, therefore various resistance genes can be acquired by them from non-fermentative bacteria or even from different strains of *Enterobacterales *. The genomic size ranges from 5.8 to 7.3 Mbp, with a core genome consisting of more than 4000 genes plus a variable accessory gene pool [51, 52]. *P. aeruginosa* is a tough bacterium to kill and it persists even after prolonged antibiotic treatment [53, 54]. It is recognized to encode an array of virulence factors and AMR genes that enable colonization and successful establishment of UTIs. In the MENA region there is high-level resistance to antimicrobials in Iraq (100 %), Egypt (100 %) and Saudi Arabia (88.9 %), indicating difficulties in managing UTIs secondary to MDR *P. aeruginosa* [13]. However, prior to the current study, there were no data available on the genomic diversity of *P. aeruginosa* isolates associated with CAUTIs in Egypt. Through phenotypic and genotypic characterization of such isolates collected from an Egyptian hospital over a 3 month period, we have demonstrated that MDR (Table 3), high-risk clones of *P. aeruginosa* are present in this clinical setting. We have also identified the presence of a pBT2436-like megaplasmid in an Egyptian isolate of *P. aeruginosa *.

*P. aeruginosa* high-risk clones are disseminated worldwide and are common causative agents of HAIs. A common feature of high-risk clones is their ability to express β-lactamases and metallo-β-lactamases. The emergence of MDR *P. aeruginosa* is considered to be a significant public health issue [55]. MDR, internationally important *P. aeruginosa* high-risk clones include ST111, ST175, ST233, ST235, ST277, ST357, ST654 and ST773 [56]. We identified eight different STs among the CAUTI isolates characterized in this study, including the high-risk clones ST357 (*n*=4) and ST773 (*n*=7), neither of which has been reported previously in Egypt (Table 4). The only previously reported ST in tertiary care Egyptian hospitals for *Pseudomonas* was ST233 (wound, sputum, urine and ear-swab samples), found to encode *NDM-1* and/or *VIM-2* by PCR [57, 58]. Our ST357 isolates (P16, P25, P31 and P32) were predicted to encode perfect sequence matches to the class C and D β-lactamases *PDC-11* and *OXA-846*, respectively. None was MDR based on phenotypic analysis, but they all showed susceptibility with increased exposure to the β-lactams [i.e. penicillin (piperacillin–tazobactam), cephalosporins (cefepime, ceftazidime), monobactam (aztreonam) and carbapenems (doripenem, meropenem)] tested (Fig. 2). The seven ST773 isolates (P5, P8, P14, P20, P26, P27 and P30) were all predicted to encode perfect matches to *PDC-16* and *OXA-395*, with all except P5 also encoding a perfect match to the metallo-β-lactamase *NDM-1*; isolates P5, P20, P26 and P30 were considered to be MDR based on EUCAST testing (Fig. 2, Table 3).

While PubMLST did not report data for ST357 in the MENA region (Table 5), this ST has been reported in Qatar (bloodstream infections, clinical isolates), Lebanon (clinical infections), Bahrain (clinical isolates) and Saudi Arabia (bacteraemia, clinical isolates) [59–63]. ST773 has only previously been reported as a clone disseminated in a burns unit in Iran [64]. Based on data available from PubMLST, ST357 has only once before been associated with UTIs (Table 4), while this study is the first to report ST773 associated with a CAUTI. Our ST data have been deposited in the PubMLST database to add to information available from the MENA region and to facilitate tracking of clinically important *P. aeruginosa* isolates contributing to infections (Table 5).

Many factors are responsible for the inherent antimicrobial resistance of *P. aeruginosa *: a large and adaptable genome, mobile genetic elements, a cell wall with low permeability and the ability of the bacterium to form biofilms [65]. Megaplasmids (plasmids >350 kbp in *Pseudomonas* [66]) are of emerging interest in the context of clinical infections associated with *P. aeruginosa *, as they have been found in nosocomial populations, are often self-transmitting and can encode a range of virulence and AMR genes [67]. Plasmid pBT2436, although >420 kbp in size, can transmit multiple resistance determinants at high efficiency [38]. We identified a pBT2436-like megaplasmid (pP9Me1, 422 938 bp) within the genome of isolate P9 (ST3765). None of the other ST3765 isolates (P11, P15, P29) we characterized harboured pBT2436-like megaplasmids and nor did any of our other isolates based on blastn and read mapping analyses (Fig. 4a). pP9Me1 encoded a range of virulence factors (*pilD*, *chpA*, *pilG*, *csrA*). Isolate P9 was determined to be a strong biofilm-former by phenotypic analysis; whether virulence genes encoded by pP9Me1 contribute to this phenotype will be the subject of future work. Similar to other pBT2436-like megaplasmids [38], pP9Me1 encoded a range of AMR genes; the most notable of these was *OXA-520*, which belongs to the *OXA-10* family of class D β-lactamases and has not been reported in Egypt previously. While included in the CARD RGI database we have been unable to find *Pseudomonas* reports on *OXA-520* in Egypt, but it has reported in the Netherlands [68, 69].

Along with the megaplasmid pP9Me1, we identified a novel plasmid (pP9Me2, 49,064 bp) within the genome of isolate P9. This smaller plasmid is predicted to encode several putative conjugation genes. Whether pP9Me1 is transmissible and pP9Me2 contributes to this transmissibility will be the subject of future studies.

Complete *Pseudomonas* plasmid sequences deposited with NCBI Genome were searched for genes homologous to core protein sequences from pBT2436 using a combination of blastn-based (Table 6), average nucleotide (Fig. S4) and phylogenetic analyses (Fig. 5). We identified another 24 pBT2436-like megaplasmids and have extended the range over which they have been found: in addition to these plasmids having been detected in Thailand, PR China, Portugal, Switzerland [38] and Egypt (this study), they can be found in the USA (*n*=2), the Netherlands (*n*=1) and France (*n*=1) (Table 6). To date, pBT2436-like megaplasmids have been detected in urine (*n*=3), CAUTIs (*n*=2) and UTIs (*n*=1) in PR China, France and Egypt (Tables 2 and 6).

Efflux pumps are of great concern with respect to the emergence of AMR in *P. aeruginosa* [70, 71]. Empirical therapy refers to the initiation of treatment before the results of diagnostic tests (such as bacterial culture and susceptibility testing) are available. When it comes to UTIs caused by *Pseudomonas* spp., empirical therapy can be challenging because of the potential for multidrug resistance among these bacteria. In Egypt, empirical therapy for UTIs typically includes the use of fluoroquinolones (ciprofloxacin and levofloxacin) [72, 73]. These antibiotics are broad spectrum and have good activity against *Pseudomonas *, although nearly 40 % of isolates in our study were resistant to ciprofloxacin. Other antibiotics such as cephalosporins (ceftazidime) and aminoglycosides (tobramycin) can also can be used [74]. It is also important to note that empirical therapy should only be used as a temporary measure, and that definitive therapy should be based on the results of bacterial culture and susceptibility testing. The choice of antimicrobial therapy should be guided by spectrum and susceptibility patterns of the aetiological pathogens, tolerability and adverse reactions, costs and availability.

Our study showed 22.5 % resistance to cephalosporins among the 31 isolates characterized, but a higher resistance was observed with quinolones (Fig. 1). This high resistance associated with quinolones is due to antibiotic misuse by patients, as these medicines are easily bought without prescription in Egypt [75]. Comparing the antimicrobial susceptibility seen in this study with that in other countries in the MENA region, ciprofloxacin demonstrated high resistance in Bahrain (100 %), Tunisia (100 %), Qatar (91.2 %), Libya (91 %), Egypt (70 %), Jordan (50.9 %), Yemen (35.7 %), Lebanon (27 %), Iraq (22.7 %), Saudia Arabia (18.1 %) and Oman (15 %). The third- and fourth-generation antipseudomonal cephalosporins demonstrated exceptionally high resistance within MDR *P. aeruginosa* clinical isolates in Qatar (96.6 %), Bahrain (86 %), Tunisia (70 %), Egypt (68 %), Libya (66 %), Yemen (47.1 %) and Iraq (41.2 %) [13]. As shown in Fig. 1b, AMR among isolates from a range of Egyptian studies showed a mean percentage of 81 % for penicillins, 79 % for cephalosporins, 77 % for others, 70 % for aminoglycosides, 61 % for quinolones, 58 % for monobactams and 37 % for carbapenems. High AMR rates against antibiotics were seen in reports that mainly focused on MDR and β-lactamase-producing strains.

Susceptibility with increased exposure was seen for 90 % (doripenem) and 87 % (piperacillin–tazobactam and aztreonam) of our isolates (Table S2). The ‘I’ susceptibility category was devised so patients infected by intermediate susceptible bacteria would be treated with a high dose of the relevant drug [76]. MexAB-OprM is a multidrug efflux protein expressed in *P. aeruginosa *. MexA is the membrane fusion protein, MexB is the inner membrane transporter and OprM is the outer membrane channel [77]. Four active efflux pumps may be responsible for an increased (2- to 16-fold) resistance to fluoroquinolones when overexpressed; namely, MexAB-OprM, MexXY/OprM, MexCD-OprJ and MexEF-OprN [78–80]. Other efflux systems MexHI-OpmD and MexPQ-OpmE have also been reported to export fluoroquinolones in *P. aeruginosa* [81, 82]. In our study, as shown in Fig. 2, all isolates harboured multiple genes responsible for the mentioned efflux pump systems. Overexpression of efflux pumps could be the leading cause of MDR in bacteria as it leads to a decreased intracellular concentration of antibiotics and reduced susceptibility to antimicrobial agents due to continuous expelling of structurally unrelated drugs [83].

Genotypic detection of resistance determinants revealed that all isolates were predicted to encode numerous AMR genes (Fig. 2) associated with resistance to aminoglycosides [*AAC(6′)-Ib4*, *AAC(6′)-Ib9*, *aadA11*, *aadA2*, *ANT(2′′)-Ia*, *ANT(3′′)-IIa*, *APH(3′)-Ia*, *APH(3′′)-Ib*, *APH(3′)-IIb*, *APH(6)-Id*], β-lactamases (*NDM-1*, *PDC-3*, *PDC-5*, *PDC-11*, *PDC-14*, *PDC-16*, *OXA-50*, *OXA-395*, *OXA-494*, *OXA-520*, *OXA-846*, *OXA-847*, *OXA-903*, *OXA-914*), fluoroquinolones (*gyrA*, *qnrVC1*), fosfomycin (*fosA*), sulfonamides (*sul1, sul2*), tetracyclines [*tet(C), tet(D*)] and chloramphenicol (*cmlA5, cmlA9, mexM, mexN, catB7*). However, resistance determinants mentioned in previous Egyptian reports, namely *AmpC*, *IMP* and *VIM* [84–86], were not detected in the current study. While the β-lactamases *OXA-2*, *OXA-4*, *OXA-10*, *OXA-50*, *OXA-486* and *PDC-3* have been reported for *P. aeruginosa* from urine, intensive care unit-associated infections, and general infections in Egypt, Saudia Arabia and Qatar [62, 87, 88], the current study is the first to report the presence of *OXA-395*, *OXA-494*, *OXA-520* (discussed above), *OXA-846*, *OXA-847*, *OXA-903*, *OXA-914*, *PDC-5*, *PDC-11*, *PDC-14* and *PDC-16* in *P. aeruginosa* in Egypt.

There are discrepancies in the literature when comparing genomic and phenotypic data for *Pseudomonas* spp. and other bacteria contributing to infections. In a recent study, the highest discordance between predicted AMR genes and phenotypic resistance profiles was observed with *P. aeruginosa* isolates (*n*=21; 9 antimicrobials, 189 combinations) rather than other *Enterobacterales* or Gram-positive bacteria [46]; 44.4 % of the results for the *P. aeruginosa* isolates showed discordance between phenotype and genotype. A third (63/189) of discordant results were major errors and 11.1 % (21/189) were very major errors. Worth mentioning is that 11 of the *P. aeruginosa* isolates showing discordant results were isolated from urine [46]. Another recent study showed that isolates recovered from urine produced the greatest discordance between genomic and phenotypic data for AMR profiles of both *Enterobacterales* and *P. aeruginosa *. Clinical implications could be drastic if hospitals are relying on ‘*susceptibility of one carbapenem to confer susceptibility to another carbapenem*’ when interpreting data [89].

It is known that the quality of the sequence data used, and the choice of AMR database/software and interpretation of these data contribute to discrepancies in AMR gene prediction [90]. The largest contributors to discrepant concordance/discordance results at the single genome level are sequence quality, read depth and the choice of reference AMR gene database, with sequencer type and DNA library preparation method having little effect on closely related gene variants and the inference of resistance phenotype [90]. It is recommended that the expected size of the genome be >90 % by comparison with a reference genome, and sequenced at ≥30× coverage. All genomes assembled for this study have >99 % completeness and >30× coverage (Table S1). There was a significant correlation (0.45, *P* value=0.01; Pearson, two-sided) between the number of AMR genes detected and number of antibiotics the strains were resistant to. There was no significant correlation (Pearson, two-sided) between the number of observed discordant results and the N50 values for genomes (correlation=0.06, *P* value=0.73) or the number of discordant results and the number of contigs contributing to genomes (correlation=0.28, *P* value=0.12). There was no significant correlation (Pearson, two-sided) between the number of virulence factors and AMR genes a genome encoded (correlation=−0.26, *P* value=0.16) or the number of virulence factors and the number of antibiotics the strains were resistant to (correlation −0.16, *P* value=0.38). In a study examining the virulence- and AMR-associated phenotypes of 302 *P *. *aeruginosa* isolates, there was no significant difference between MDR and non-MDR isolates with respect to their expression of virulence factors, with the exception of pyocyanin production [91]. Similarly, our previous phenotypic work (*n*=103 *P. aeruginosa* isolates) found no associations between AMR and biofilm formation [14].

We suggest that our high discordance level (i.e. major errors WGS-R/DDT-S; 68.1 %) may be accounted for by the pooling of ‘S’ and ‘I’ isolates together into one category in accordance with the EUCAST update for susceptibility definitions in 2019. Because of these new definitions and breakpoints, *P. aeruginosa* becomes intrinsically less susceptible to an antimicrobial, and will thus rarely reach the S susceptible category. Infections require increased exposure for almost all antimicrobials to be treated, hence *P. aeruginosa* phenotypes fall into the clinical category of ‘susceptible with increased exposure’ (i.e. I) for all relevant antimicrobials (except meropenem) [92]. An in-depth review of genotype–phenotype AMR concordance was performed by the EUCAST subcommittee, which concluded that promising high levels of concordance were noted for certain bacterial groups (* Enterobacteriaceae* and staphylococci), while other species (* P. aeruginosa* and *Acinetobacter baumannii *) proved much more difficult to interpret [93]. The major challenge for *P. aeruginosa* and *A. baumannii* lies in the identification or prediction of resistance due to chromosomal alterations resulting in modification of expression levels, particularly with respect to efflux pumps, outer membrane proteins and intrinsic β-lactamases.

**Fig. 6.**
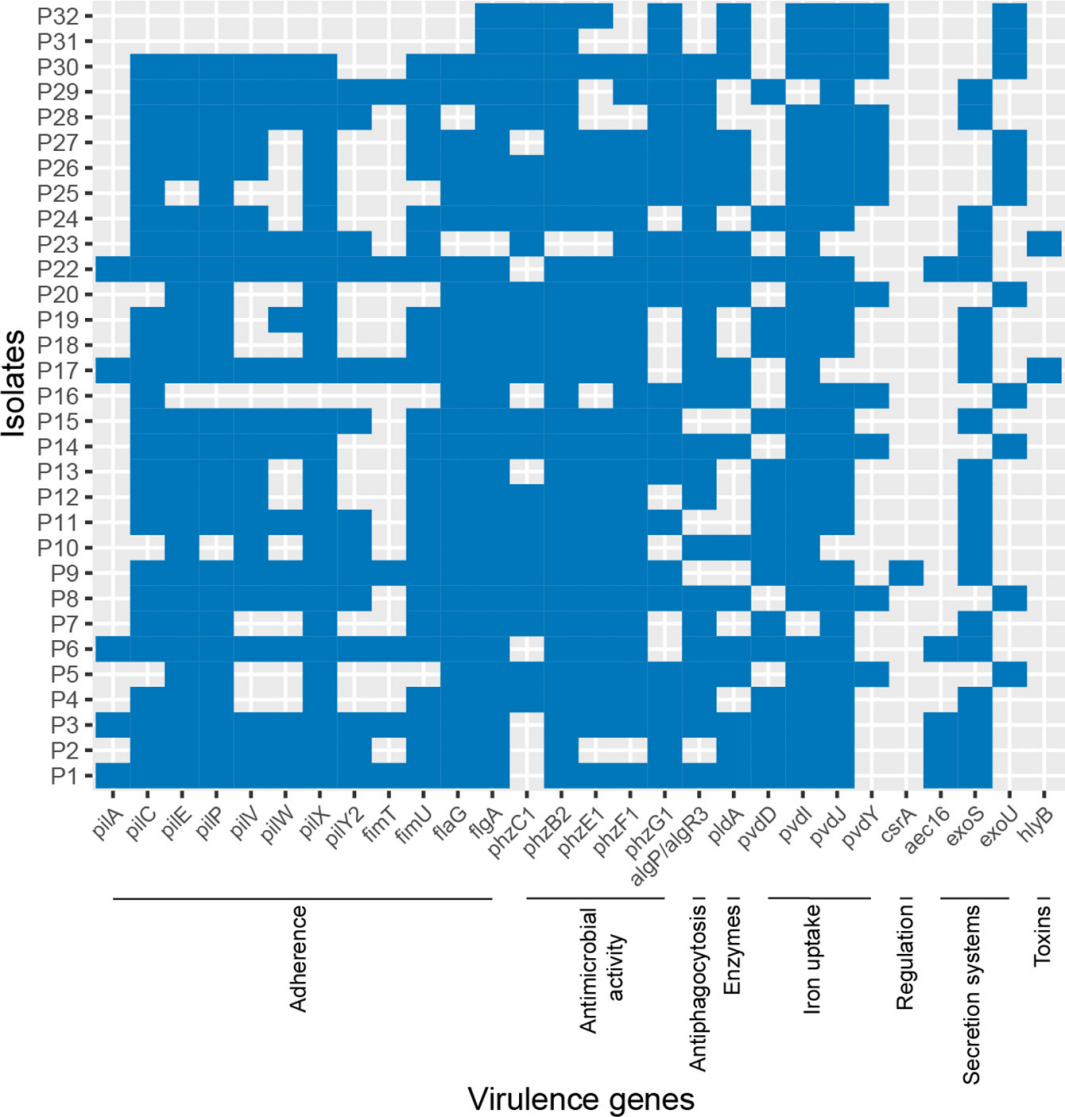
Prevalence of virulence factors (<100 % presence) predicted to be encoded within the genomes of the 31 *P. aeruginosa* isolates using the VFDB. Adherence: *pilA*, *fimT*, *pilY2*, *pilW*, *pilV*, *fimU*, *pilC*, *flaG*, *pilE*, *pilP*, *pilX*, *flgA*. Antimicrobial activity: *phzC1*, *phzG1F1*, *phzB2*. Antiphagocytosis: *algP*/*algR3*. Enzymes: *pldA*. Iron uptake: *pvdY*, *pvdD*, *pvdJ*, *pvdI*. Regulation: *csrA*. Secretion systems: *aec16*, *exoU*, *exoS*. Toxins: *hlyB*.

For many bacteria, the urinary tract represents a harsh, nutrient-limited environment; thus, to survive and grow within the urinary tract, *P. aeruginosa* produces toxins and proteases that injure the host tissue to release nutrients, while also providing a niche for bacterial invasion and dissemination [94]. As shown in Fig. 6 and mentioned in Table S3, our isolates encoded genes predicted to produce proteases, toxins, quorum sensing and secretion systems. The main traits of the virulence genes predicted to be encoded by the isolates characterized in this study were related to adherence and secretion systems, signifying that the isolates could be biofilm-producers, as suggested by a previous report [95]. The process of biofilm formation in *P. aeruginosa* is complex and multifactorial, involving the coordination of many different genes, including those encoding for motility, quorum sensing, alginate production and regulation systems [96].

In comparison with a previous report [97], a total of 220 virulence genes were found among their *Pseudomonas* biofilm-forming isolates by comparing their WGS and VFDB data. All the isolates were able to produce biofilm. The most-represented groups of virulence genes identified among the isolates’ genomes were those for flagellar protein synthesis (17 %), type III secretion system (T3SS) machinery (17.7 %), type IV pili-related functions and twitching motility (14.5 %), and alginate biosynthesis and regulation (12 %). In our study, a total of 215 of virulence genes (Table S3) were found, with most of our isolates forming a strong biofilm (Fig. 3). The most represented groups of virulence genes identified were those associated with flagellar protein synthesis (22.3 %), T3SS (18.5 %), type IV pili and twitching motility (14.8 %), and alginate biosynthesis and regulation (12.1 %).

*pilA* and *fimT* have previously been reported as biofilm-associated genes [98, 99]. Another report showed that MDR biofilm-forming *P. aeruginosa* ST111 encoded both *pilA* and *fimT*, but these genes were absent from the ST235 pangenome [100]. In our study, *pilA* and *fimT* genes were predicted to be encoded in the genomes of the strong biofilm-formers (P1, P3, P17, P22) and one of the moderate biofilm-formers (P6). The *fimT* gene was found without *pilA* in isolates P9 and P29, which were strong and weak biofilm-formers, respectively. T3SS genes *exoT* and *exoY* were found in all isolates, whereas *exoS* and *exoU* were not found concurrently in our isolates; *exoU* ^+^ isolates were P5, P8, P14, P16, P20, P25, P26, P27, P30, P31 and P32, while *exoS* ^+^ isolates were P1, P2, P3, P4, P6, P7, P9, P10, P11, P12, P13, P15, P17, P18, P19, P22, P23, P24, P28 and P29 (Fig. 4). In general, *Pseudomonas* encoding *exoS* show an invasive phenotype, while those isolates encoding *exoU* are cytotoxic in nature [101]. *exoS* and e*xoU* are generally mutually exclusive, although some studies have reported rare isolates harbouring both exotoxins [102, 103].

## Conclusions

This study demonstrates the utility of next-generation sequencing to define the diversity of AMR and virulence elements, and highlights STs of *P. aeruginosa* contributing to CAUTIs in Egypt. This information is valuable in furthering the design of diagnostics and therapeutics for the treatment of *P. aeruginosa* infections in the MENA region. Continuous monitoring and surveillance programmes should be encouraged in Egypt to track new high-risk clones and to analyse the emergence of new clones as well as novel resistance determinants.

## Supplementary Data

Supplementary material 1

Supplementary material 2

## References

[1] Haque M, Sartelli M, McKimm J, Abu Bakar M (2018). Health care-associated infections - an overview. Infect Drug Resist.

[2] Tan CW, Chlebicki MP (2016). Urinary tract infections in adults. Singapore Med J.

[3] Feneley RCL, Hopley IB, Wells PNT (2015). Urinary catheters: history, current status, adverse events and research agenda. J Med Eng Technol.

[4] Kalsi J, Arya M, Wilson P, Mundy A (2003). Hospital-acquired urinary tract infection. Int J Clini Pract.

[5] Mestrovic T, Robles Aguilar G, Swetschinski LR, Ikuta KS, Gray AP (2022). The burden of bacterial antimicrobial resistance in the WHO European region in 2019: a cross-country systematic analysis. The Lancet Public Health.

[6] Lamas Ferreiro JL, Álvarez Otero J, González González L, Novoa Lamazares L, Arca Blanco A (2017). *Pseudomonas aeruginosa* urinary tract infections in hospitalized patients: mortality and prognostic factors. PLoS One.

[7] ECDC (2022). Antimicrobial resistance in the EU/EEA (EARS-Net)-Annual epidemiological report for 2021. https://www.ecdc.europa.eu/en/publications-data/surveillance-antimicrobial-resistance-europe-2021.

[8] WHO & ECDC (2022). Antimicrobial resistance surveillance in Europe 2022 - 2020 data. https://www.ecdc.europa.eu/en/publications-data/antimicrobial-resistance-surveillance-europe-2022-2020-data.

[9] WHO (2017). WHO publishes list of bacteria for which new antibiotics are urgently needed. https://www.who.int/news/item/27-02-2017-who-publishes-list-of-bacteria-for-which-new-antibiotics-are-urgently-needed.

[10] Morales E, Cots F, Sala M, Comas M, Belvis F (2012). Hospital costs of nosocomial multi-drug resistant *Pseudomonas aeruginosa* acquisition. BMC Health Serv Res.

[11] CDC (2019). Antibiotic resistance threats report by centers for disease control and prevention. https://www.cdc.gov/drugresistance/biggest-threats.html.

[12] Ramadan R, Omar N, Dawaba M, Moemen D (2021). Bacterial biofilm dependent catheter associated urinary tract infections: characterization, antibiotic resistance pattern and risk factors. Egyptian J Basic Appl Sci.

[13] Al-Orphaly M, Hadi HA, Eltayeb FK, Al-Hail H, Samuel BG (2021). Epidemiology of multidrug-resistant *Pseudomonas aeruginosa* in the middle East and North Africa Region. mSphere.

[14] Eladawy M, El-Mowafy M, El-Sokkary MMA, Barwa R (2021). Antimicrobial resistance and virulence characteristics in ERIC-PCR typed biofilm forming isolates of *P. aeruginosa*. Microb Pathog.

[15] Merritt JH, Kadouri DE, O’Toole GA (2005). Growing and analyzing static biofilms. Curr Protoc Microbiol.

[16] Stepanovic S, Vukovic D, Dakic I, Savic B, Svabic-Vlahovic M (2000). A modified microtiter-plate test for quantification of staphylococcal biofilm formation. J Microbiol Methods.

[17] Hayward MR, Petrovska L, Jansen VAA, Woodward MJ (2016). Population structure and associated phenotypes of *Salmonella enterica* serovars Derby and Mbandaka overlap with host range. BMC Microbiol.

[18] Newberry F, Shibu P, Smith-Zaitlik T, Eladawy M, McCartney AL (2023). Lytic bacteriophage vB_KmiS-Kmi2C disrupts biofilms formed by members of the *Klebsiella* oxytoca complex, and represents a novel virus family and genus. J Appl Microbiol.

[19] Bolger AM, Lohse M, Usadel B (2014). Trimmomatic: a flexible trimmer for Illumina sequence data. Bioinformatics.

[20] Bankevich A, Nurk S, Antipov D, Gurevich AA, Dvorkin M (2012). SPAdes: a new genome assembly algorithm and its applications to single-cell sequencing. J Comput Biol.

[21] Kolmogorov M, Yuan J, Lin Y, Pevzner PA (2019). Assembly of long, error-prone reads using repeat graphs. Nat Biotechnol.

[22] Vaser R, Sović I, Nagarajan N, Šikić M (2017). Fast and accurate de novo genome assembly from long uncorrected reads. Genome Res.

[23] Huang YT, Liu PY, Shih PW (2021). Homopolish: a method for the removal of systematic errors in nanopore sequencing by homologous polishing. Genome Biol.

[24] Wick RR, Holt KE (2022). Polypolish: short-read polishing of long-read bacterial genome assemblies. PLoS Comput Biol.

[25] Zimin AV, Salzberg SL (2020). The genome polishing tool POLCA makes fast and accurate corrections in genome assemblies. PLoS Comput Biol.

[26] Hu J, Fan J, Sun Z, Liu S, Berger B (2020). NextPolish: a fast and efficient genome polishing tool for long-read assembly. Bioinformatics.

[27] Bushnell B (2014). BBMap: a fast, accurate, splice-aware aligner. https://www.osti.gov/biblio/1241166.

[28] Parks DH, Imelfort M, Skennerton CT, Hugenholtz P, Tyson GW (2015). CheckM: assessing the quality of microbial genomes recovered from isolates, single cells, and metagenomes. Genome Res.

[29] Jain C, Rodriguez-R LM, Phillippy AM, Konstantinidis KT, Aluru S (2018). High throughput ANI analysis of 90K prokaryotic genomes reveals clear species boundaries. Nat Commun.

[30] Chun J, Oren A, Ventosa A, Christensen H, Arahal DR (2018). Proposed minimal standards for the use of genome data for the taxonomy of prokaryotes. Int J Syst Evol Microbiol.

[31] Schwengers O, Jelonek L, Dieckmann MA, Beyvers S, Blom J (2021). Bakta: rapid and standardized annotation of bacterial genomes via alignment-free sequence identification. Microb Genom.

[32] Chen L, Yang J, Yu J, Yao Z, Sun L (2005). VFDB: a reference database for bacterial virulence factors. Nucleic Acids Res.

[33] Curran B, Jonas D, Grundmann H, Pitt T, Dowson CG (2004). Development of a multilocus sequence typing scheme for the opportunistic pathogen *Pseudomonas aeruginosa*. J Clin Microbiol.

[34] Jolley KA, Bray JE, Maiden MCJ (2018). Open-access bacterial population genomics: BIGSdb software, the PubMLST.org website and their applications. Wellcome Open Res.

[35] McArthur AG, Waglechner N, Nizam F, Yan A, Azad MA (2013). The comprehensive antibiotic resistance database. Antimicrob Agents Chemother.

[36] Asnicar F, Thomas AM, Beghini F, Mengoni C, Manara S (2020). Precise phylogenetic analysis of microbial isolates and genomes from metagenomes using PhyloPhlAn 3.0. Nat Commun.

[37] Parks DH, Chuvochina M, Waite DW, Rinke C, Skarshewski A (2018). A standardized bacterial taxonomy based on genome phylogeny substantially revises the tree of life. Nat Biotechnol.

[38] Cazares A, Moore MP, Hall JPJ, Wright LL, Grimes M (2020). A megaplasmid family driving dissemination of multidrug resistance in *Pseudomonas*. Nat Commun.

[39] Martin M (2011). Cutadapt removes adapter sequences from high-throughput sequencing reads. EMBnet J.

[40] Li H (2013). Aligning sequence reads, clone sequences and assembly contigs with BWA-MEM. arXiv arXiv.

[41] Li H, Handsaker B, Wysoker A, Fennell T, Ruan J (2009). The sequence alignment/map format and SAMtools. Bioinformatics.

[42] Giménez M, Ferrés I, Iraola G (2022). Improved detection and classification of plasmids from circularized and fragmented assemblies. bioRxiv.

[43] Rognes T, Flouri T, Nichols B, Quince C, Mahé F (2016). VSEARCH: a versatile open source tool for metagenomics. PeerJ.

[44] Steinegger M, Söding J (2017). MMseqs2 enables sensitive protein sequence searching for the analysis of massive data sets. Nat Biotechnol.

[45] Stamatakis A (2014). RAxML version 8: a tool for phylogenetic analysis and post-analysis of large phylogenies. Bioinformatics.

[46] Rebelo AR, Bortolaia V, Leekitcharoenphon P, Hansen DS, Nielsen HL (2022). One day in Denmark: comparison of phenotypic and genotypic antimicrobial susceptibility testing in bacterial isolates from clinical settings. Front Microbiol.

[47] Vanstokstraeten R, Piérard D, Crombé F, De Geyter D, Wybo I (2023). Genotypic resistance determined by whole genome sequencing versus phenotypic resistance in 234 *Escherichia coli* isolates. Sci Rep.

[48] Bowers RM, Kyrpides NC, Stepanauskas R, Harmon-Smith M, Doud D (2017). Minimum information about a single amplified genome (MISAG) and a metagenome-assembled genome (MIMAG) of bacteria and archaea. Nat Biotechnol.

[49] Redondo-Salvo S, Bartomeus-Peñalver R, Vielva L, Tagg KA, Webb HE (2021). COPLA, a taxonomic classifier of plasmids. BMC Bioinformatics.

[50] Li Y, Zhu Y, Zhou W, Chen Z, Moran RA (2022). Alcaligenes faecalis metallo-β-lactamase in extensively drug-resistant *Pseudomonas aeruginosa* isolates. Clin Microbiol Infect.

[51] Arnold M, Wibberg D, Blom J, Schatschneider S, Winkler A (2015). Draft genome sequence of *Pseudomonas aeruginosa* strain WS136, a highly cytotoxic ExoS-positive wound isolate recovered from pyoderma gangrenosum. Genome Announc.

[52] Klockgether J, Cramer N, Wiehlmann L, Davenport CF, Tümmler B (2011). *Pseudomonas aeruginosa* genomic structure and diversity. Front Microbiol.

[53] Cottalorda A, Dahyot S, Soares A, Alexandre K, Zorgniotti I (2022). Phenotypic and genotypic within-host diversity of *Pseudomonas aeruginosa* urinary isolates. Sci Rep.

[54] Cottalorda A, Leoz M, Dahyot S, Gravey F, Grand M (2020). Within-host microevolution of *Pseudomonas aeruginosa* urinary isolates: a seven-patient longitudinal genomic and phenotypic study. Front Microbiol.

[55] Angeletti S, Cella E, Prosperi M, Spoto S, Fogolari M (2018). Multi-drug resistant *Pseudomonas aeruginosa* nosocomial strains: molecular epidemiology and evolution. Microb Pathog.

[56] Kocsis B, Gulyás D, Szabó D (2021). Diversity and distribution of resistance markers in *Pseudomonas aeruginosa* international high-risk clones. Microorganisms.

[57] Zafer MM, Al-Agamy MH, El-Mahallawy HA, Amin MA, El Din Ashour S (2015). Dissemination of VIM-2 producing *Pseudomonas aeruginosa* ST233 at tertiary care hospitals in Egypt. BMC Infect Dis.

[58] Zafer MM, Amin M, El Mahallawy H, Ashour M-D, Al Agamy M (2014). First report of NDM-1-producing *Pseudomonas aeruginosa* in Egypt. Int J Infect Dis.

[59] Alamri AM, Alfifi S, Aljehani Y, Alnimr A (2020). Whole genome sequencing of ceftolozane-tazobactam and ceftazidime-avibactam resistant *Pseudomonas aeruginosa* isolated from a blood stream infection reveals VEB and chromosomal metallo-beta lactamases as genetic determinants: a case report. Infect Drug Resist.

[60] Bitar I, Salloum T, Merhi G, Hrabak J, Araj GF (2022). Genomic characterization of mutli-drug resistant *Pseudomonas aeruginosa* clinical isolates: evaluation and determination of ceftolozane/tazobactam activity and resistance mechanisms. Front Cell Infect Microbiol.

[61] Sid Ahmed MA, Abdel Hadi H, Abu Jarir S, Ahmad Khan F, Arbab MA (2022). Prevalence and microbiological and genetic characteristics of multidrug-resistant *Pseudomonas aeruginosa* over three years in Qatar. Antimicrob Steward Healthc Epidemiol.

[62] Sid Ahmed MA, Khan FA, Sultan AA, Söderquist B, Ibrahim EB (2020). β-lactamase-mediated resistance in MDR-*Pseudomonas aeruginosa* from Qatar. Antimicrob Resist Infect Control.

[63] Zowawi HM, Syrmis MW, Kidd TJ, Balkhy HH, Walsh TR (2018). Identification of carbapenem-resistant *Pseudomonas aeruginosa* in selected hospitals of the gulf cooperation council states: dominance of high-risk clones in the region. J Med Microbiol.

[64] Yousefi S, Nahaei MR, Farajnia S, Aghazadeh M, Iversen A (2013). A multiresistant clone of *Pseudomonas aeruginosa* sequence type 773 spreading in a burn unit in Orumieh, Iran. APMIS.

[65] Lambert PA (2002). Mechanisms of antibiotic resistance in *Pseudomonas aeruginosa*. J R Soc Med.

[66] Hall JPJ, Botelho J, Cazares A, Baltrus DA (2022). What makes a megaplasmid?. Phil Trans R Soc B.

[67] Urbanowicz P, Bitar I, Izdebski R, Baraniak A, Literacka E (2021). Epidemic territorial spread of IncP-2-Type VIM-2 carbapenemase-encoding megaplasmids in nosocomial *Pseudomonas aeruginosa* populations. Antimicrob Agents Chemother.

[68] Croughs PD, Klaassen CHW, van Rosmalen J, Maghdid DM, Boers SA (2018). Unexpected mechanisms of resistance in Dutch *Pseudomonas aeruginosa* isolates collected during 14 years of surveillance. Int J Antimicrob Agents.

[69] Del Barrio-Tofiño E, López-Causapé C, Oliver A (2020). *Pseudomonas aeruginosa* epidemic high-risk clones and their association with horizontally-acquired β-lactamases: 2020 update. Int J Antimicrob Agents.

[70] Blanco P, Hernando-Amado S, Reales-Calderon JA, Corona F, Lira F (2016). Bacterial multidrug efflux pumps: much more than antibiotic resistance determinants. Microorganisms.

[71] Kishk RM, Abdalla MO, Hashish AA, Nemr NA, El Nahhas N (2020). Efflux *MexAB*-mediated resistance in *P. aeruginosa* isolated from patients with healthcare associated infections. Pathogens.

[72] Abdelkhalik AM, Agha MM, Zaki AM, Tahoun AT (2018). Clinical and lab-assessed antibiotic resistance pattern of uropathogens among women with acute uncomplicated cystitis. Egypt J Hosp Med.

[73] Nouh K, Kasem A, Shaher H, Elawady H, Gomaa R (2021). The Egyptian Urological Guidelines, First Edition, Chapter XI: Urinary Tract Infections Guidelines.

[74] Moustafa BH, Rabie MM, El Hakim IZ, Badr A, El Balshy M (2021). Egyptian pediatric clinical practice guidelines for urinary tract infections in infants and children (evidence based). Egypt Pediatric Association Gaz.

[75] Ramadan AA, Abdelaziz NA, Amin MA, Aziz RK (2019). Novel blaCTX-M variants and genotype-phenotype correlations among clinical isolates of extended spectrum beta lactamase-producing *Escherichia coli*. Sci Rep.

[76] Rodloff A, Bauer T, Ewig S, Kujath P, Müller E (2008). Susceptible, intermediate, and resistant – the intensity of antibiotic action. Deutsches Arzteblatt Int.

[77] Tsutsumi K, Yonehara R, Ishizaka-Ikeda E, Miyazaki N, Maeda S (2019). Structures of the wild-type MexAB-OprM tripartite pump reveal its complex formation and drug efflux mechanism. Nat Commun.

[78] Köhler T, Michéa-Hamzehpour M, Henze U, Gotoh N, Curty LK (1997). Characterization of MexE-MexF-OprN, a positively regulated multidrug efflux system of *Pseudomonas aeruginosa*. Mol Microbiol.

[79] Masuda N, Sakagawa E, Ohya S, Gotoh N, Tsujimoto H (2000). Substrate specificities of MexAB-OprM, MexCD-OprJ, and MexXY-OprM efflux pumps in *Pseudomonas aeruginosa*. Antimicrob Agents Chemother.

[80] Zhang L, Li X-Z, Poole K (2001). Fluoroquinolone susceptibilities of efflux-mediated multidrug-resistant *Pseudomonas aeruginosa*, *Stenotrophomonas maltophilia* and *Burkholderia cepacia*. J Antimicrob Chemother.

[81] Mima T, Sekiya H, Mizushima T, Kuroda T, Tsuchiya T (2005). Gene cloning and properties of the RND-type multidrug efflux pumps MexPQ-OpmE and MexMN-OprM from *Pseudomonas aeruginosa*. Microbiol Immunol.

[82] Sekiya H, Mima T, Morita Y, Kuroda T, Mizushima T (2003). Functional cloning and characterization of a multidrug efflux pump, mexHI-opmD, from a *Pseudomonas aeruginosa* mutant. Antimicrob Agents Chemother.

[83] Nikaido H, Pagès JM (2012). Broad-specificity efflux pumps and their role in multidrug resistance of Gram-negative bacteria. FEMS Microbiol Rev.

[84] Abbas HA, El-Ganiny AM, Kamel HA (2018). Phenotypic and genotypic detection of antibiotic resistance of *Pseudomonas aeruginosa* isolated from urinary tract infections. Afr Health Sci.

[85] Basha AM, El-Sherbiny GM, Mabrouk MI (2020). Phenotypic characterization of the Egyptian isolates “extensively drug-resistant *Pseudomonas aeruginosa*” and detection of their metallo-β-lactamases encoding genes. Bull Natl Res Cent.

[86] El-Domany RA, Emara M, El-Magd MA, Moustafa WH, Abdeltwab NM (2017). Emergence of imipenem-resistant *Pseudomonas aeruginosa* clinical isolates from Egypt coharboring VIM and IMP carbapenemases. Microb Drug Resist.

[87] Al-Agamy MH, Jeannot K, El-Mahdy TS, Samaha HA, Shibl AM (2016). Diversity of molecular mechanisms conferring carbapenem resistance to *Pseudomonas aeruginosa* isolates from Saudi Arabia. Can J Infect Dis Med Microbiol.

[88] El-Shouny WA, Ali SS, Sun J, Samy SM, Ali A (2018). Drug resistance profile and molecular characterization of extended spectrum beta-lactamase (ESβL)-producing *Pseudomonas aeruginosa* isolated from burn wound infections. Essential oils and their potential for utilization. Microb Pathog.

[89] Ku PM, Hobbs DA, Gilmore M, Hobbs AL (2021). 1234. can susceptibility to one carbapenem be conferred to another? frequency of discordance in gram-negative clinical isolates. Open Forum Infect Dis.

[90] Doyle RM, O’Sullivan DM, Aller SD, Bruchmann S, Clark T (2020). Discordant bioinformatic predictions of antimicrobial resistance from whole-genome sequencing data of bacterial isolates: an inter-laboratory study. Microb Genom.

[91] Gajdács M, Baráth Z, Kárpáti K, Szabó D, Usai D (2021). No correlation between biofilm formation, virulence factors, and antibiotic resistance in *Pseudomonas aeruginosa*: results from a laboratory-based in vitro study. Antibiotics.

[92] Nabal Díaz SG, Algara Robles O, García-Lechuz Moya JM (2022). New definitions of susceptibility categories EUCAST 2019: clinic application. Rev Esp Quimioter.

[93] Ellington MJ, Ekelund O, Aarestrup FM, Canton R, Doumith M (2017). The role of whole genome sequencing in antimicrobial susceptibility testing of bacteria: report from the EUCAST Subcommittee. Clin Microbiol Infect.

[94] Flores-Mireles AL, Walker JN, Caparon M, Hultgren SJ (2015). Urinary tract infections: epidemiology, mechanisms of infection and treatment options. Nat Rev Microbiol.

[95] Datar R, Coello Pelegrin A, Orenga S, Chalansonnet V, Mirande C (2021). Phenotypic and genomic variability of serial peri-lung transplantation *Pseudomonas aeruginosa* isolates from cystic fibrosis patients. Front Microbiol.

[96] Thi MTT, Wibowo D, Rehm BHA (2020). *Pseudomonas aeruginosa* biofilms. Int J Mol Sci.

[97] Díaz-Ríos C, Hernández M, Abad D, Álvarez-Montes L, Varsaki A (2021). New sequence type ST3449 in multidrug-resistant *Pseudomonas aeruginosa* isolates from a cystic fibrosis patient. Antibiotics.

[98] Deligianni E, Pattison S, Berrar D, Ternan NG, Haylock RW (2010). *Pseudomonas aeruginosa* cystic fibrosis isolates of similar RAPD genotype exhibit diversity in biofilm forming ability in vitro. BMC Microbiol.

[99] Sultan M, Arya R, Kim KK (2021). Roles of two-component systems in *Pseudomonas aeruginosa* virulence. Int J Mol Sci.

[100] Redfern J, Wallace J, van Belkum A, Jaillard M, Whittard E (2021). Biofilm associated genotypes of multiple antibiotic resistant *Pseudomonas aeruginosa*. BMC Genomics.

[101] Karthikeyan RS, Priya JL, Leal SM, Toska J, Rietsch A (2013). Host response and bacterial virulence factor expression in *Pseudomonas aeruginosa* and *Streptococcus pneumoniae* corneal ulcers. PLoS One.

[102] Rodrigues YC, Furlaneto IP, Maciel AHP, Quaresma AJPG, de Matos ECO (2020). High prevalence of atypical virulotype and genetically diverse background among *Pseudomonas aeruginosa* isolates from a referral hospital in the Brazilian Amazon. PLoS ONE.

[103] Sarges E do SNF, Rodrigues YC, Furlaneto IP, de Melo MVH, Brabo GL da C (2020). *Pseudomonas aeruginosa* type III secretion system virulotypes and their association with clinical features of cystic fibrosis patients. Infect Drug Resist.

[104] Botelho J, Grosso F, Quinteira S, Mabrouk A, Peixe L (2017). The complete nucleotide sequence of an IncP-2 megaplasmid unveils a mosaic architecture comprising a putative novel blaVIM-2-harbouring transposon in *Pseudomonas aeruginosa*. J Antimicrob Chemother.

[105] Li M, Guan C, Song G, Gao X, Yang W (2022). Characterization of a conjugative multidrug resistance IncP-2 megaplasmid, pPAG5, from a clinical *Pseudomonas aeruginosa* isolate. Microbiol Spectr.

[106] Xiong J, Alexander DC, Ma JH, Déraspe M, Low DE (2013). Complete sequence of pOZ176, a 500-kilobase IncP-2 plasmid encoding IMP-9-mediated carbapenem resistance, from outbreak isolate *Pseudomonas aeruginosa* 96. Antimicrob Agents Chemother.

[107] Zheng D, Wang X, Wang P, Peng W, Ji N (2016). Genome sequence of *Pseudomonas citronellolis* SJTE-3, an estrogen- and polycyclic aromatic hydrocarbon-degrading bacterium. Genome Announc.

[108] Schmid M, Frei D, Patrignani A, Schlapbach R, Frey JE (2018). Pushing the limits of de novo genome assembly for complex prokaryotic genomes harboring very long, near identical repeats. Nucleic Acids Res.

[109] Yuan M, Chen H, Zhu X, Feng J, Zhan Z (2017). Psy153-MDR, a P12969-DIM-related MEGA plasmid carrying Bla(IMP-45) and armA, from clinical *Pseudomonas putida*. Oncotarget.

[110] Abd El-Baky RM, Masoud SM, Mohamed DS, Waly NG, Shafik EA (2020). Prevalence and some possible mechanisms of colistin resistance among multidrug-resistant and extensively drug-resistant *Pseudomonas aeruginosa*. Infect Drug Resist.

[111] Abdel-Rhman SH, Rizk DE (2018). Serotypes, antibiogram and genetic relatedness of *Pseudomonas aeruginosa* isolates from urinary tract infections at urology and nephrology center, Mansoura, Egypt. AiM.

[112] Abou-Dobara MI, Deyab MA, Elsawy EM, Mohamed HH (2010). Antibiotic susceptibility and genotype patterns of *Escherichia coli*, *Klebsiella pneumoniae* and *Pseudomonas aeruginosa* isolated from urinary tract infected patients. Pol J Microbiol.

[113] Ahmed O, Mohamed H, Salem W, Afifi M, Song Y (2021). Efficacy of ethanolic extract of *Syzygium aromaticum* in the treatment of multidrug-resistant *Pseudomonas aeruginosa* clinical isolates associated with urinary tract infections. Evid Based Complement Alternat Med.

[114] Edward EA, El Shehawy MR, Abouelfetouh A, Aboulmagd E (2023). Prevalence of different virulence factors and their association with antimicrobial resistance among *Pseudomonas aeruginosa* clinical isolates from Egypt. BMC Microbiol.

[115] El-Mahdy R, El-Kannishy G (2019). Virulence factors of carbapenem-resistant *Pseudomonas aeruginosa* In hospital-acquired infections in Mansoura, Egypt. Infect Drug Resist.

[116] El-Mokhtar MA, Hassanein KM, Ahmed AS, Gad GF, Amin MM (2020). Antagonistic activities of cell-free supernatants of lactobacilli against extended-spectrum β-lactamase producing *Klebsiella pneumoniae* and *Pseudomonas aeruginosa*. Infect Drug Resist.

[117] El Shamy AA, Zakaria Z, Tolba MM, Salah Eldin N, Rabea A-T (2021). *AmpC β*-lactamase variable expression in common multidrug-resistant nosocomial bacterial pathogens from a tertiary hospital in Cairo, Egypt. Int J Microbiol.

[118] Elbargisy RM (2022). Characterization of uropathogenic *Pseudomonas aeruginosa*: serotypes, resistance phenotypes, and virulence genotypes. J Pure Appl Microbiol.

[119] Elnegery AA, Mowafy WK, Zahra TA, Abou El-Khier NT (2021). Study of quorum-sensing LasR and RhlR genes and their dependent virulence factors in *Pseudomonas aeruginosa* isolates from infected burn wounds. Access Microbiol.

[120] Farhan SM, Ibrahim RA, Mahran KM, Hetta HF, Abd El-Baky RM (2019). Antimicrobial resistance pattern and molecular genetic distribution of metallo-β-lactamases producing *Pseudomonas aeruginosa* isolated from hospitals in Minia, Egypt. Infect Drug Resist.

[121] Gad GF, el-Domany RA, Ashour HM (2008). Antimicrobial susceptibility profile of *Pseudomonas aeruginosa* isolates in Egypt. J Urol.

[122] Gad GF, Mohamed HA, Ashour HM (2011). Aminoglycoside resistance rates, phenotypes, and mechanisms of Gram-negative bacteria from infected patients in upper Egypt. PLoS One.

[123] Hamza EH, El-Shawadfy AM, Allam AA, Hassanein WA (2023). Study on pyoverdine and biofilm production with detection of LasR gene in MDR *Pseudomonas aeruginosa*. Saudi J Biol Sci.

[124] Hashem H, Hanora A, Abdalla S, Shawky A, Saad A (2016). Carbapenem susceptibility and multidrug-resistance in *Pseudomonas aeruginosa* isolates in Egypt. Jundishapur J Microbiol.

[125] Mohamed WF, Askora AA, Mahdy MMH, El-Hussieny EA, Abu-Shady HM (2022). Isolation and characterization of bacteriophages active against *Pseudomonas aeruginosa* strains isolated from diabetic foot infections. Arch Razi Inst.

[126] Nassar O, Desouky SE, El-Sherbiny GM, Abu-Elghait M (2022). Correlation between phenotypic virulence traits and antibiotic resistance in Pseudomonas aeruginosa clinical isolates. Microb Pathog.

[127] Mohamed S, Marwa A, Hamada H, Amro H (2016). Mutations in -lactamases detected in multidrug resistant gram negative bacteria isolated from community acquired urinary tract infections in Assiut, Egypt. Afr J Microbiol Res.

[128] Shaaban M, Al-Qahtani A, Al-Ahdal M, Barwa R (2017). Molecular characterization of resistance mechanisms in *Pseudomonas aeruginosa* isolates resistant to carbapenems. J Infect Dev Ctries.

[129] Zafer MM, Al-Agamy MH, El-Mahallawy HA, Amin MA, Ashour MSE-D (2014). Antimicrobial resistance pattern and their beta-lactamase encoding genes among *Pseudomonas aeruginosa* strains isolated from cancer patients. Biomed Res Int.

[130] Kuepper J, Ruijssenaars HJ, Blank LM, de Winde JH, Wierckx N (2015). Complete genome sequence of solvent-tolerant *Pseudomonas putida* S12 including megaplasmid pTTS12. J Biotechnol.

[131] Scheetz MH, Hoffman M, Bolon MK, Schulert G, Estrellado W (2009). Morbidity associated with *Pseudomonas aeruginosa* bloodstream infections. Diagn Microbiol Infect Dis.

[132] Eltoukhy A, Jia Y, Lamraoui I, Abo-Kadoum MA, Atta OM (2022). Transcriptome analysis and cytochrome P450 monooxygenase reveal the molecular mechanism of Bisphenol A degradation by *Pseudomonas putida* strain YC-AE1. BMC Microbiol.

[133] Chung H, Merakou C, Schaefers MM, Flett KB, Martini S (2022). Rapid expansion and extinction of antibiotic resistance mutations during treatment of acute bacterial respiratory infections. Nat Commun.

[134] Zhang X, Wang L, Li D, Li P, Yuan L (2021). An Incp-2 plasmid sublineage associated with dissemination of Bla(IMP-45) among carbapenem-resistant *Pseudomonas aeruginosa*. Emerg Microbes Infect.

[135] Zhu Y, Chen J, Shen H, Chen Z, Yang QW (2021). Emergence of ceftazidime- and avibactam-resistant *Klebsiella pneumoniae* carbapenemase-producing *Pseudomonas aeruginosa* in China. mSystems.

[136] Long X, Wang X, Mao D, Wu W, Luo Y (2022). A novel XRE-type regulator mediates phage lytic development and multiple host metabolic processes in *Pseudomonas aeruginosa*. Microbiol Spectr.

[137] Zhang B, Xu X, Song X, Wen Y, Zhu Z (2022). Emerging and re-emerging KPC-producing hypervirulent *Pseudomonas aeruginosa* ST697 and ST463 between 2010 and 2021. Emerg Microbes Infect.

[138] Fang Y, Baloch Z, Zhang W, Hu Y, Zheng R (2022). Emergence of carbapenem-resistant ST244, ST292, and ST2446 *Pseudomonas aeruginosa* clones in burn patients in Yunnan province. Infect Drug Resist.

[139] Gao J, Wei X, Yin L, Jin Y, Bai F (2022). Emergence and transfer of plasmid-harbored rmtB in a clinical multidrug-resistant *Pseudomonas aeruginosa* strain. Microorganisms.

[140] Chen M, Cai H, Li Y, Wang N, Zhang P (2022). Plasmid-borne AFM alleles in *Pseudomonas aeruginosa* clinical isolates from China. Microbiol Spectr.

[141] Hu Y, Liu C, Wang Q, Zeng Y, Sun Q (2021). Emergence and expansion of a carbapenem-resistant *Pseudomonas aeruginosa* clone are associated with plasmid-borne *bla*
_KPC-2_ and virulence-related genes. mSystems.

